# From Analytical Profiling to Liposomal Delivery: Cannabinol as a Model for Antioxidant Encapsulation and Diffusion Enhancement

**DOI:** 10.3390/molecules30163433

**Published:** 2025-08-20

**Authors:** Aleksandar Marinković, Đura Nakarada, Miloš Marinković, Hadi Waisi, Vladislav Živanić, Arcadio Vazquez, Miloš Mojović

**Affiliations:** 1 Institute of General and Physical Chemistry, Studentski trg 12/V, 11158 Belgrade, Serbia; amarinkovic@iofh.bg.ac.rs (A.M.); milosmarinkovic83@gmail.com (M.M.); hadiwaisi@yahoo.com (H.W.); 2 Center for Physical Chemistry of Biological Systems, BioScope Labs, Faculty of Physical Chemistry, University of Belgrade, Studentski trg 12-16, 11158 Belgrade, Serbia; 3 BS BG Technology, Nikolaja Gogolja 24, 11030 Belgrade, Serbia; v.zivanic@bsbg.eu; 4 Mimexis Pharma S.R.O., V doline 1515/1b, 101 00 Praha, Czech Republic; arcadiovalvarez@gmail.com

**Keywords:** cannabinol, liposomes, antioxidant activity, EPR spectroscopy, diffusion

## Abstract

This study explores the antioxidant potential and delivery performance of five structurally distinct cannabinoids, with a particular focus on cannabinol (CBN). Comprehensive structural characterization using mass spectrometry (MS) and nuclear magnetic resonance (NMR) revealed key molecular features relevant to antioxidant function. Among the tested compounds, CBN exhibited the most potent and balanced radical scavenging activity against 2,2-diphenyl-1-picrylhydrazyl (DPPH), hydroxyl, and superoxide radicals. Based on these findings, CBN was selected for formulation into soy lecithin liposomes. The resulting CBN-loaded liposomes displayed favorable colloidal properties, with an average size of approximately 122.9 ± 0.4 nm. Results indicating increased membrane order upon CBN incorporation suggest enhanced stability of the liposomal bilayer. Antioxidant activity assays showed that CBN-loaded liposomes retain significant radical scavenging capacity, though with a moderate reduction compared to free CBN. EPR imaging further demonstrated superior diffusion of liposomal CBN through a gelatin-based semi-solid model compared to the control solution. While the current model does not replicate skin architecture, it provides a cost-effective and reproducible platform for early-stage screening of formulation mobility. These results position CBN-loaded liposomes as a promising candidate for dermal antioxidant applications, combining favorable physicochemical properties with enhanced diffusion behavior.

## 1. Introduction

Oxidative stress is a key driver of skin aging and a major contributor to various dermatological conditions. Triggered by ultraviolet (UV) radiation, pollutants, and chronic inflammation, excessive production of reactive oxygen species (ROS) overwhelms the skin’s antioxidant defenses, disrupting redox homeostasis and damaging key biomolecules such as lipids, collagen, elastin, and DNA [1,2]. These molecular alterations compromise the skin barrier, accelerate aging, and increase susceptibility to inflammatory and neoplastic disorders. To mitigate oxidative damage, topical antioxidants have been widely explored in both dermatological and cosmetic applications. Compounds such as polyphenols, vitamins (C and E), and terpenoids have demonstrated potential as radical scavengers [3]. However, their clinical translation is often limited by instability, low skin permeability, or even pro-oxidant activity under certain conditions (e.g., high concentrations or presence of metal ions) [4,5]. These challenges have stimulated the search for novel antioxidant agents with enhanced stability, efficacy, and skin penetration profiles [6,7,8]. Recent advances in lipid–polymer hybrid systems have demonstrated enhanced delivery efficiency and physicochemical stability for poorly soluble bioactives [9], while plant-derived antioxidants such as hesperidin [10] and Ayurvedic extracts [11] have shown promising dermatological and antioxidant properties, underscoring their potential in modern topical formulations.

Cannabinoids have recently gained significant attention for their multifunctional biological activities, including antioxidant, anti-inflammatory, antitumor, and antifibrotic effects [12]. Both plant-derived (phytocannabinoids) and synthetic cannabinoids show promise in combating oxidative stress, with several outperforming conventional antioxidants in vitro and in vivo. Δ^9^-tetrahydrocannabinol (THC), for instance, has demonstrated neuroprotective and antioxidant effects comparable to or greater than vitamin E [13,14]. Cannabidiol (CBD), a non-psychoactive cannabinoid, has been reported to be more potent than ascorbate or α-tocopherol in ROS neutralization, an effect attributed to its two phenolic hydroxyl groups [13,14]. Beyond CBD and THC, other cannabinoids such as cannabidiolic acid (CBDA), cannabigerol (CBG), and cannabinol (CBN) have shown significant antioxidant and anti-inflammatory properties. CBG and CBD were found to reduce ROS levels in human dermal fibroblasts more effectively than vitamin C [15,16], while CBG also exhibited strong anti-inflammatory activity by inhibiting cytokine release induced by UV or chemical stimuli. CBN has been shown to modulate inflammatory responses in keratinocytes by downregulating pro-inflammatory cytokines (e.g., IL-8, IL-12, and IL-31) and upregulating IL-10, suggesting both direct and indirect antioxidant effects [17]. Despite their promising bioactivity, the topical application of cannabinoids faces several formulation challenges. These include poor aqueous solubility, high lipophilicity (e.g., CBD has a log P ≈ 5–7), low skin permeability due to the stratum corneum barrier, and chemical instability (e.g., oxidation of CBD under light and heat) [13,18,19,20]. These factors hinder consistent dermal absorption and therapeutic dosing, which is further complicated by interindividual variations in skin properties and microbiome, as well as regulatory constraints surrounding cannabinoid-based formulations [21]. 

**Figure 1:**
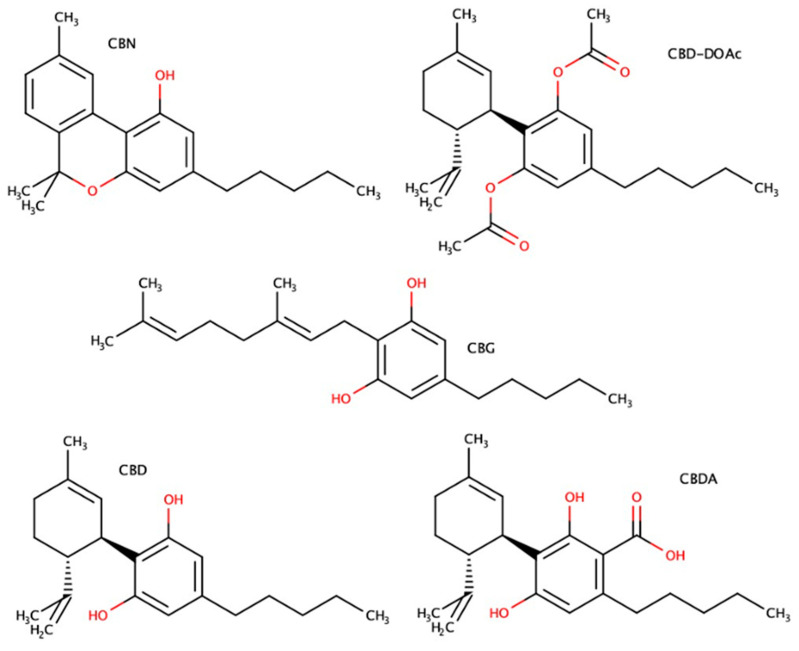
Chemical structures of the cannabinoids analyzed in this study: cannabinol (CBN) (6,6,9-Trimethyl-3-pentyl-benzo[c]chromen-1-ol); cannabidiol diacetate (CBD-DOAc) ([3-acetyloxy-2-[(1R,6R)-3-methyl-6-prop-1-en-2-ylcyclohex-2-en-1-yl]-5-pentylphenyl] acetate); cannabidiolic acid (CBDA) (2,4-dihydroxy-3-[(1R,6R)-3-methyl-6-prop-1-en-2-ylcyclohex-2-en-1-yl]-6-pentylbenzoic acid); cannabigerol (CBG) (2-[(2E)-3,7-Dimethylocta-2,6-dienyl]-5-pentylbenzene-1,3-diol); and cannabidiol (CBD) (2-[(1R,6R)-6-isopropenyl-3-methylcyclohex-2-en-1-yl]-5-pentylbenzene-1,3-diol).

To overcome these obstacles, advanced strategies have been developed, including prodrug derivatization and nanocarrier-based delivery systems. Acetylated CBD derivatives, such as CBD diacetate, improve lipophilicity and stability while retaining biological activity after enzymatic conversion in vivo [18]. Nanocarriers like liposomes, nanoemulsions, ethosomes, and solid lipid nanoparticles enhance cannabinoid solubility, protect labile compounds from degradation, and facilitate deeper skin penetration [19,22,23,24]. Liposomes are particularly attractive due to their ability to encapsulate both hydrophilic and lipophilic molecules, high biocompatibility, and compatibility with skin lipids. Flexible liposomes (e.g., ethosomes) further improve transdermal delivery via enhanced vesicle deformability and lipid fluidization of the stratum corneum [24]. Continued investigation into new delivery strategies and therapeutic profiles is essential to fully harness the use of cannabinoids in skin care and treatment. In this study, we compared the antioxidant potential of five structurally distinct cannabinoids—CBN, CBD-DOAc, CBDA, CBG, and CBD (Figure 1)—to identify the compound with the most potent and well-balanced radical scavenging activity. The cannabinoid showing the highest antioxidant potential was then encapsulated into soy lecithin liposomes. The resulting formulation underwent physicochemical characterization, antioxidant activity evaluation, and diffusion testing in a gelatin-based semi-solid model, selected as a rapid and cost-effective tool for early-stage comparative assessment of formulation mobility.

## 2. Results and Discussion

### 2.1. GC–MS Purity and Thermal Behavior of Cannabinoid Standards

Each cannabinoid reference material (CBN, CBD-DOAc, CBDA, CBG, CBD) was analyzed in an individual GC–MS run under identical conditions. In all cases, the total-ion chromatogram (TIC) showed a single analyte peak integrating to 100% of the TIC area within the reporting window, with no detectable impurities above the 0.5% TIC threshold. To accommodate minor integration uncertainty, we conservatively report each material as >99% pure by GC–MS. Similar GC-based purity assessments and the impact of injector temperature on cannabinoid stability have been discussed previously [25,26,27].

When injected alone, the CBDA standard yielded a single peak at a retention time (RT) of approximately 15.46 min, which was numerically identical to the RT observed for the CBD standard. The accompanying mass spectrum matched that of CBD rather than CBDA, confirming in-inlet decarboxylation of CBDA → CBD under the applied GC conditions. Thermal degradation of cannabinoid acids in GC injection ports is well documented [27,28,29] and may lead to erroneous identification unless derivatization or alternative methods are employed [26,28,29].

Because GC–MS detects only the decarboxylated product, CBDA identity was confirmed independently by NMR. Diagnostic features included the carboxylic carbon at δC 175.2 ppm and downfield phenolic signals not present in CBD. This underscores the analytical importance of recognizing temperature-driven cannabinoid transformations during GC analysis.

A summary of retention times and GC–MS purity results is provided in Table 1.

Specifically, the molecular ion peak (M^+^) at *m*/*z* 314 and the characteristic base peak at *m*/*z* 231, and fragment ions at *m*/*z* 299, 246, 193, and 174 matched those of neutral CBD in both samples (CBD and CBDA), as previously reported in cannabinoid MS libraries. No unique acid-derived fragments (e.g., *m*/*z* 358 for undegraded CBDA) were observed in the CBDA sample.

### 2.2. NMR Data

Samples identified as single components (>99% TIC area; Section 2.1) were subsequently analyzed by NMR spectroscopy to confirm their identity and purity. The recorded spectra matched previously reported literature data for all tested compounds. In the case of CBDA, the presence of a carboxyl group was confirmed by the signal of the carboxyl carbon at δC 175.2 ppm.

Cannabinol (CBN)

^1^H NMR (400 MHz, CDCl_3_, 298 K) δ 8.17 (s, 1H, Ar-H), 7.15–7.05 (m, 2H, Ar-H), 6.44 (s, 1H), 6.26 (s, 1H), 5.25 (br s, 1H, phenolic OH), 2.50–2.38 (m, 5H), 1.60–1.57 (m, 8H, overlapping 2 × Me + CH_2_), 1.34–1.30 (m, 4H), 0.90 (t, *J* = 7.3 Hz, 3H). Data consistent with lit. [30,31,32].

^13^C NMR (100 MHz, CDCl_3_, 298 K) δ 154.8, 153.2, 144.8, 137.1 (2C), 127.8, 127.7, 126.6, 122.8, 111.0, 110.1, 108.9, 77.6 (C-7, overlaps CDCl_3_), 35.8, 31.7, 30.7, 27.3 (2C), 22.7, 21.7, 14.2. Data consistent with lit. [30,31,32].

Cannabidiol diacetate (CBD-DOAc)

^1^H NMR (399.74 MHz, CDCl_3_, 298 K) δ 6.69 (s, 2H, Ar-H), 5.18 (br s, 1H, olefinic H-2), 4.54–4.43 (m, 2H, =CH_2_ H-9a/H-9b), 3.51–3.47 (m, 1H, CH–O), 2.66–2.51 (m, 3H), 2.18–2.10 (m, 4H), 2.04–1.98 (s, 6H, 2 × OAc CH_3_), 1.80–1.71 (m, 2H), 1.69–1.65 (s, 3H, isoprenyl Me), 1.61–1.53 (m, 3H), 1.33–1.24 (m, 4H), 0.88–0.84 (t, *J* ≈ 7 Hz, 3H). Data consistent with lit. [25,26,33].

^13^C NMR (100.52 MHz, CDCl_3_, 298 K) δ 169.2 (2C, 2 × OAc C=O), 149.8 (2C), 148.0, 142.2, 133.1, 126.1, 124.7, 121.2 (2C), 111.2, 45.8, 38.6, 35.4, 31.7, 30.6, 30.5, 28.9, 23.6, 22.7, 21.1 (2C), 19.8, 14.2. Data consistent with lit. [25,26,33].

Cannabidiolic acid (CBDA)

^1^H NMR (399.74 MHz, CDCl_3_, 298 K) δ 8.39 (br s, exch., ~1H, CO_2_H/phenolic; variably observed), 6.26 (s, 1H, Ar-H), 5.23 (s, 1H, olefinic H-2), 4.50–4.43 (m, 2H, =CH_2_ H-9a/H-9b), 4.07–4.04 (m, 1H, CH-1), 3.04 (m, 1H), 2.90–2.86 (m, 2H, benzylic), 2.23–1.96 (m, aliphatic CH_2_/CH near ring), 1.77–1.71 (m, 2H), 1.68–1.55 (ovrlp m, 2 × Me + adjacent CH; ~6H total by integration), 1.34–1.31 (m, 4H, pentyl CH_2_), 0.89–0.86 (t, *J* ≈ 7 Hz, 3H, pentyl CH_3_). Data consistent with lit. [30,31,34].

^13^C NMR (100.52 MHz, CDCl_3_, 298 K) δ 175.2 (C=O, CO_2_H), 165.5, 161.3, 150.0, 146.8, 133.2, 126.9, 126.5, 116.2, 111.4, 110.7 (2C), 104.5, 45.4, 37.3, 36.9, 32.9, 32.5, 31.4, 30.5, 23.8, 23.3, 19.5, 14.5 (2C). Minor closely spaced resonances likely reflect conformers/rotamers in solution; assignments in agreement with published CBDA data. Data consistent with lit. [30,31,34].

Cannabigerol (CBG)

^1^H NMR (400 MHz, CDCl_3_, 298 K) δ 6.23 (s, 2H, Ar-H-3/5), 5.28–5.24 (m, 1H, olefinic), 5.09–5.03 (m, 3H, =CH/=CH_2_ region), 3.39–3.37 (m, 2H, benzylic CH_2_), 2.45–2.42 (m, 2H), 2.11–2.02 (m, 4H), 1.80 (s, 3H, vinylic Me), 1.67–1.66 (s, 3H, vinylic Me), 1.58–1.51 (m, overlap, allylic/side-chain CH_2_), 1.35–1.25 (m, 4H, pentyl CH_2_), 0.89–0.86 (t, *J* = 7 Hz, 3H, pentyl CH_3_). Phenolic OH signals weak/variable in CDCl_3_; not reliably observed. Data consistent with lit. [30,31].

^13^C NMR (100 MHz, CDCl_3_, 298 K) δ 155.0 (2C, Ar-C-O C-2/C-6), 143.0, 139.2, 132.3, 124.0, 121.9, 110.8, 108.6 (2C, Ar-CH C-3/C-5), 39.9, 35.7, 31.7, 31.0, 26.6, 25.9, 22.8, 22.5, 17.9, 16.4, 14.2. Data consistent with lit. [30,31].

Cannabidiol (CBD)

^1^H NMR (399.74 MHz, CDCl_3_, 298 K) δ 6.25 (s, 1H, Ar-H), 6.16 (s, 1H, Ar-H), 5.97 (br s, exch., 1H, phenolic OH), 5.56–5.55 (br s, exch., 1H, phenolic OH), 4.73 (br s, 1H, olefinic H-2), 4.65–4.54 (m, 2H, =CH_2_ H-9a/H-9b), 3.86–3.82 (m, 1H, CH-O), 2.44–2.05 (m, aliphatic CH/CH_2_ adjacent to ring), 1.84–1.74 (m, 2H), 1.64 (s, 3H, vinylic Me), 1.58–1.50 (m, 3H, allylic/side-chain overlap), 1.33–1.23 (m, 4H, pentyl CH_2_), 0.88–0.84 (t, *J* ≈ 7 Hz, 3H, pentyl CH_3_). Data consistent with lit. [30,31,35,36].

^13^C NMR (100.52 MHz, CDCl_3_, 298 K) δ 156.2, 154.1, 149.5, 143.2, 140.3, 124.4, 114.0, 110.0 (2C), 108.2, 46.4, 37.4, 35.7, 31.7, 30.8, 30.6, 28.6, 23.9, 22.7, 20.7, 14.2. Data consistent with lit. [30,31,35,36].

### 2.3. Antiradical Activity of Free Cannabinoid Compounds

**Figure 2:**
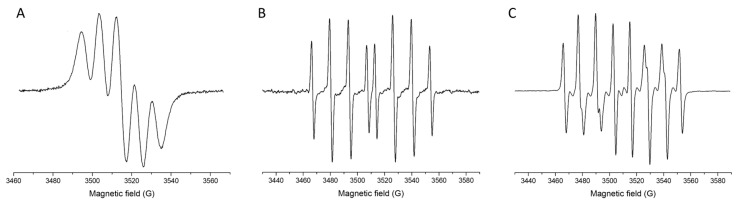
Representative EPR spectra of selected radical species or their spin-adducts: DPPH (**A**), DEPMPO/OH (**B**), and DEPMPO/OOH (**C**).

**Figure 3:**
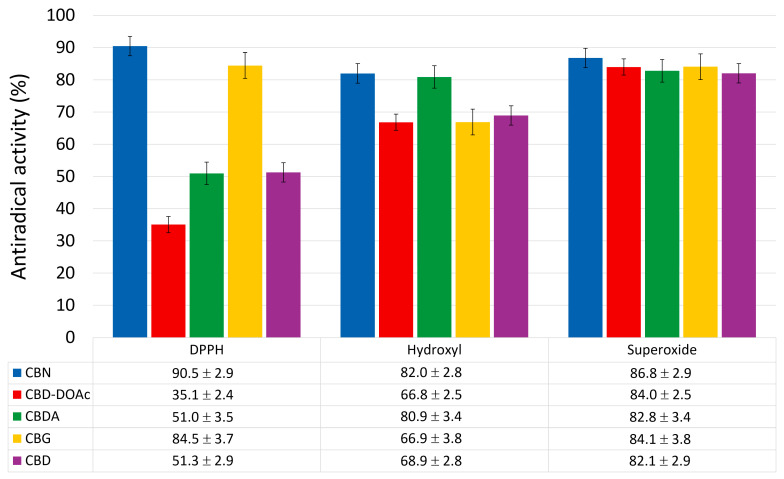
Antiradical activity (in %) of cannabinoid compounds towards selected radical species. Data are presented as mean ± SD (*n* = 3 independent experiments).

Representative EPR spectra of the measured radical species—DPPH, spin-adduct DEPMPO/OH, and spin-adduct DEPMPO/OOH—recorded under control conditions (without cannabinoids) are shown in Figure 2, providing a reference for their characteristic signal profiles used in quantification. The comparative results, summarized in Figure 3, clearly demonstrate differences in radical scavenging efficiency among the five tested cannabinoids—CBN, CBD-DOAc, CBDA, CBG, and CBD—against DPPH, hydroxyl (•OH), and superoxide anion (O_2_•^−^) radicals.

Among the tested compounds, CBN displayed the highest scavenging activity toward DPPH radicals (90.5%), followed by CBG (84.5%). This indicates strong electron or hydrogen-donating capabilities, which are important mechanisms for neutralizing DPPH, a stable nitrogen-centered radical. In contrast, CBD-DOAc demonstrated significantly weaker DPPH scavenging (35.1%), likely due to oxidative modifications that impair its ability to participate in redox reactions.

In the hydroxyl radical assay, CBN (82.0%) and CBDA (80.9%) exhibited the strongest scavenging activity, underscoring their potential in mitigating damage from highly reactive oxygen species. Interestingly, despite its good DPPH activity, CBG showed only moderate hydroxyl radical scavenging (66.9%), pointing to possible structural selectivity. CBD-DOAc once again had the weakest effect (66.8%).

Scavenging of superoxide anion radicals was more uniformly effective across all cannabinoids, with CBN achieving the highest activity (86.8%), followed by CBD-DOAc, CBG, and CBDA, all above 82%. These values suggest that most of the tested compounds interact favorably with this biologically relevant oxygen species.

Taken together, CBN consistently demonstrated strong and balanced radical scavenging across all tested radical species, making it the most promising candidate for further formulation. Accordingly, its encapsulation into liposomes was pursued to investigate whether its antioxidant activity can be maintained or enhanced in vesicle-bound form.

### 2.4. Physicochemical Characterization of Liposomes by DLS

**Figure 4:**
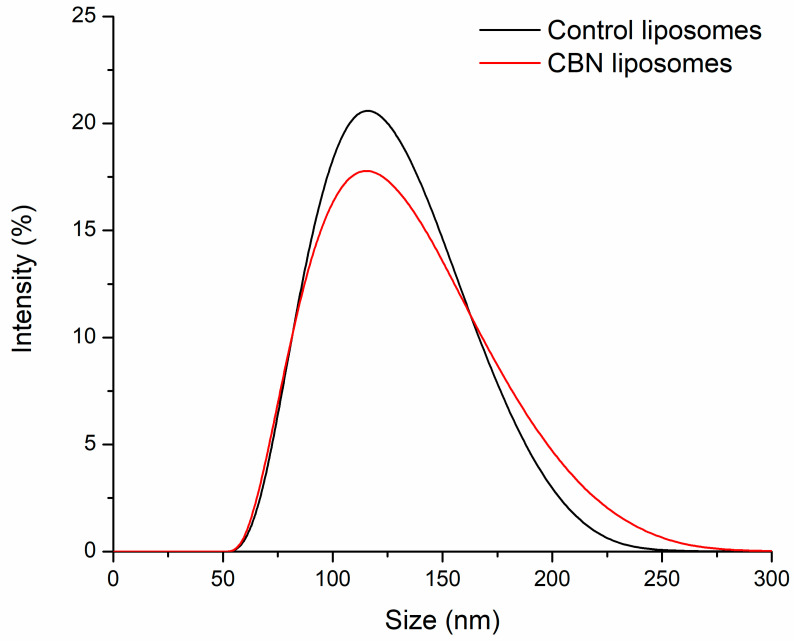
A DLS profile of CBN-containing and control liposomes.

Dynamic light scattering (DLS) was employed to evaluate the size distribution (Figure 4) and surface charge (zeta potential) of the liposomal formulations. The average hydrodynamic diameter of CBN-loaded soy lecithin liposomes was measured to be 122.9 ± 0.4 nm, indicating the formation of nanosized, likely unilamellar vesicles suitable for potential biological applications. In comparison, control liposomes prepared without CBN exhibited a slightly smaller average size of 119.7 ± 0.7 nm. The polydispersity index (PDI) of the CBN-loaded liposomes was 0.238 ± 0.003, while that of the control liposomes was 0.217 ± 0.004, indicating moderately narrow size distributions in both cases. These PDI values fall within the range (≤0.3) typically considered acceptable for reproducible nanoparticle formulations, suggesting that the incorporation of CBN did not lead to notable aggregation or heterogeneity. Notably, the size distribution of the CBN-loaded liposomes was only marginally broader than that of the control formulation, suggesting that the incorporation of CBN does not significantly compromise the uniformity or stability of the vesicle population. This slight increase in size range may reflect minor variations in bilayer packing or encapsulation efficiency but remains within a range compatible with consistent nanoparticle behavior.

Zeta potential measurements revealed that both formulations carried a surface charge of approximately −36 mV, reflecting good colloidal stability due to electrostatic repulsion between vesicles. The similarity in surface charge suggests that incorporation of CBN at 5% (*w*/*w*) does not significantly alter the overall charge distribution of the liposomal membrane.

These results confirm the successful formation of stable, uniformly sized liposomes with and without active compound, providing a suitable carrier system for further antioxidant testing and potential in vivo applications.

### 2.5. Spin Labeling EPR Analysis of Liposome Membrane Fluidity

**Figure 5:**
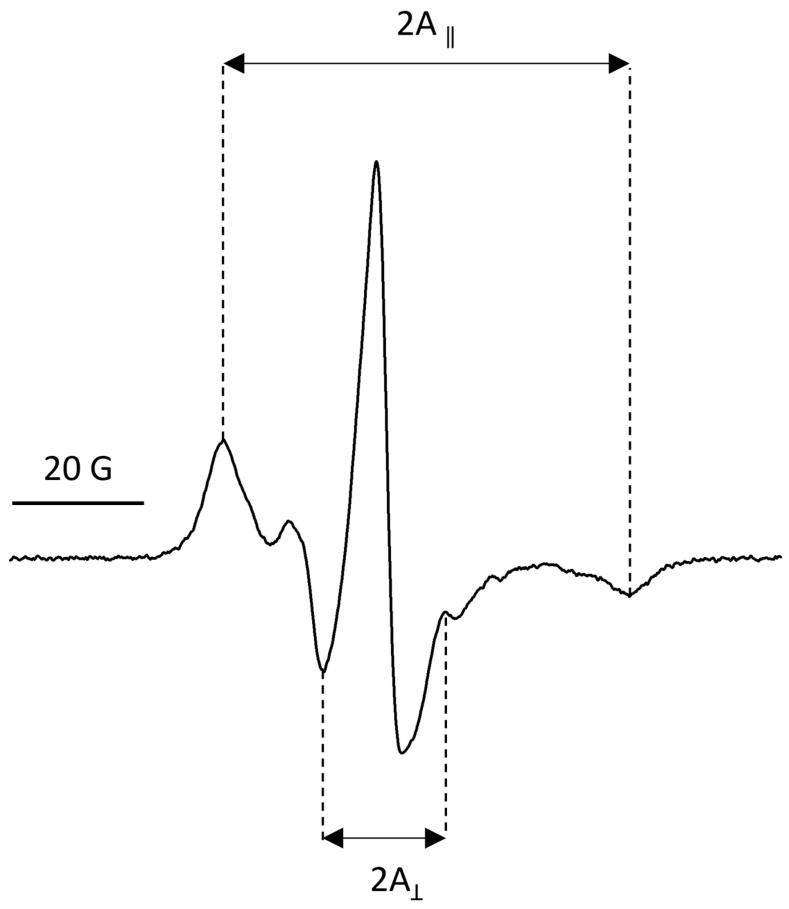
EPR spectrum of spin-label 5-DS integrated into CBN-containing liposomes. A_||_ and A_⊥_ represent outer and inner hyperfine splitting constants, respectively.

To assess the effect of CBN incorporation on membrane dynamics, electron paramagnetic resonance (EPR) spectroscopy was performed using 5-doxyl stearic acid (5-DS) as a spin label. This nitroxide-labeled fatty acid integrates near the surface of the lipid bilayer and is sensitive to changes in membrane fluidity [37]. The EPR spectral line shape (Figure 5) reflects the motion of the probe and allows quantification of the membrane order parameter (S), which ranges from 0 (completely disordered, isotropic motion) to 1 (perfectly ordered, rigid membrane). The values of S were obtained by calculating the ratio corresponding to hyperfine anisotropy, in accordance with the method outlined by [38].

The control soy lecithin liposomes exhibited an S value of 0.82 ± 0.02 (mean ± SD, *n* = 3), indicative of a moderately ordered lipid environment. In contrast, CBN-loaded liposomes showed a higher order parameter of 0.88 ± 0.01 (mean ± SD, *n* = 3; *p* < 0.05, *t*-test), suggesting increased rigidity of the bilayer. This increase in membrane order upon CBN incorporation may be attributed to interactions between CBN molecules and the phospholipid acyl chains, potentially leading to tighter packing or reduced lateral mobility within the bilayer. Such ordering effects are commonly observed with hydrophobic or amphipathic bioactive molecules that intercalate into lipid membranes and influence their physical state [37,38]. Compared to highly rigid liposomes, such as those where S approaches 1, our CBN-loaded vesicles remain highly elastic, facilitating potential stratum corneum penetration. This elasticity, combined with CBN’s highly redox-active properties, may mitigate oxidative damage to skin lipids, contributing to improved transdermal performance. Furthermore, the increased bilayer order is associated with greater vesicle stability, preventing premature drug release and improving the shelf life of liposomal formulations [39]. This finding holds significant implications for future studies aimed at developing CBN-loaded liposomes for transdermal drug delivery by optimizing lipid composition and including alternative preparation methods such as extrusion. Specifically, subsequent investigations could leverage in silico molecular dynamics simulations—building upon the model employed in this work for liposome assembly—to correlate variations in bilayer order with permeability outcomes.

### 2.6. Antiradical Activity of CBN-Loaded Liposomes

**Figure 6:**
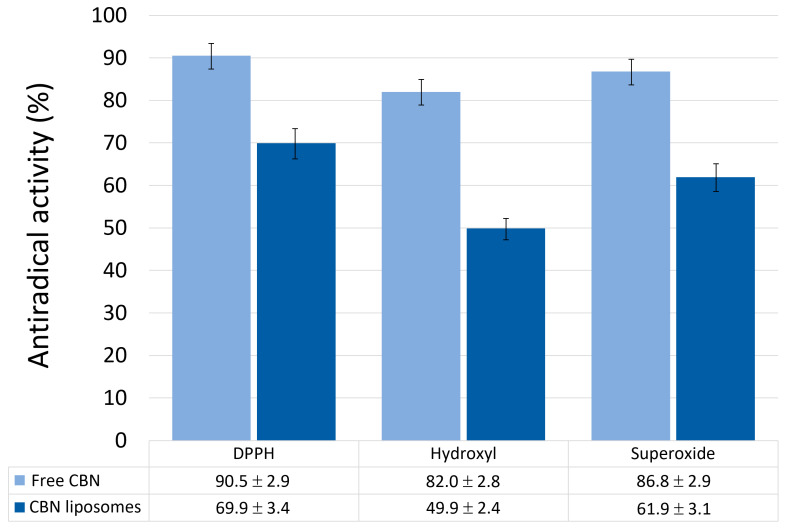
Antiradical activity (in %) of free CBN and CBN-containing liposomes towards selected radical species. Data are presented as mean ± SD (*n* = 3 independent experiments).

To assess whether encapsulation affects the antioxidant potential of CBN, liposomes containing 5% CBN and 95% soy lecithin were tested under the same conditions as the free compounds. The results (Figure 6) show that CBN-loaded liposomes retained significant antiradical activity, although it was somewhat reduced compared to non-encapsulated CBN. The scavenging activity of CBN liposomes toward DPPH radicals was 69.9%, indicating that the encapsulated form still efficiently interacts with stable free radicals. Against hydroxyl radicals, activity dropped to 49.9%, while superoxide anion scavenging was 61.9%. In all three assays, the activity of the liposomal form was lower than that of free CBN (90.5% for DPPH, 82.0% for hydroxyl, and 86.8% for superoxide), which is expected due to the partial barrier posed by the lipid bilayer and possible reduced mobility of CBN molecules. Importantly, control liposomes made solely of soy lecithin showed negligible radical scavenging across all assays in contrast to the pronounced antioxidant activity of CBN-loaded liposomes. This confirms that the observed effects stem from encapsulated CBN rather than the lipid matrix. While previous studies have reported concentration-dependent radical scavenging by soy lecithin, particularly in ABTS and DPPH assays [40], our controls exhibited minimal activity under the conditions tested. Thus, although a synergistic role of the lipid matrix cannot be completely ruled out, the antioxidant effects observed here are primarily attributable to CBN encapsulation.

These findings suggest that while some loss in immediate radical-scavenging potency may occur upon encapsulation, CBN-loaded liposomes still offer a promising strategy for delivering antioxidant activity in a more stable or controlled-release form. Further studies could investigate whether this formulation enhances stability, bioavailability, or prolonged activity in biological settings.

In dermal drug research, antioxidants such as resveratrol, curcumin, and quercetin have been extensively formulated into liposomes or related vesicular carriers to enhance stability, cutaneous absorption, and therapeutic efficacy. Liposomal encapsulation of these polyphenols has been shown to improve skin penetration and to exert protective effects against oxidative stress and photoaging in both ex vivo and in vivo settings [41,42,43,44,45]. Our observation that CBN retains broad radical-scavenging activity following encapsulation is consistent with these reports and highlights its potential to join this class of dermal antioxidants. Compared to these benchmark compounds, CBN demonstrates a particularly balanced scavenging profile toward different radical species, which may be advantageous for managing complex oxidative stress in skin. Importantly, liposomal and ethosomal formulations of cannabinoids such as cannabidiol have already demonstrated improved dermal deposition, controlled release, and anti-inflammatory efficacy [46,47,48,49], supporting the feasibility of cannabinoid-based nanocarriers for topical use. While encapsulation reduced the immediate scavenging efficiency relative to free CBN, the benefits of liposomal delivery—protection from degradation, controlled release, and enhanced cutaneous bioavailability—make CBN-loaded liposomes a promising candidate for further development in antioxidant-driven dermal applications.

### 2.7. Diffusion of CBN-Loaded Liposomes in a Hydrogel-like Environment

**Figure 7:**
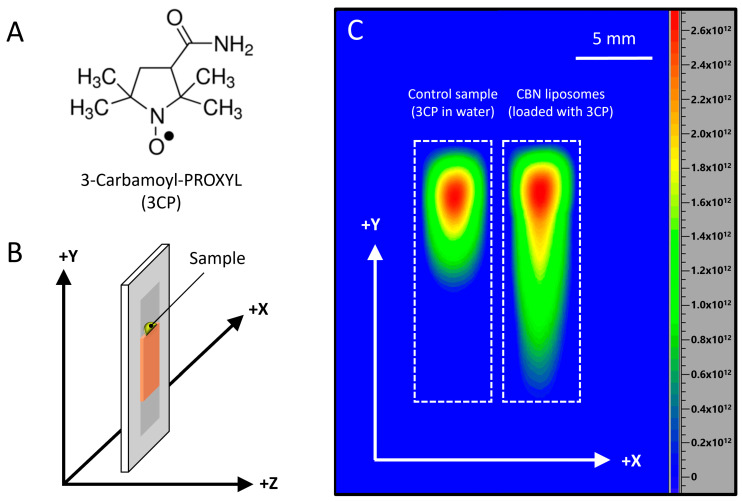
(**A**) Chemical structure of the spin probe 3-carbamoyl-PROXYL (3CP). (**B**) Schematic illustration of the experimental setup: a gelatin plate with the test sample applied on top, as positioned inside the EPRI resonator. (**C**) X-band 2D EPR image (XY plane) acquired 30 min after sample placement. The image includes two regions: the left corresponds to the control sample (aqueous solution of 3CP) and the right to CBN liposomes loaded with 3CP. Signal intensity is shown in arbitrary units using a color scale representing 3CP concentration: red indicates the highest levels, yellow and light green indicate moderate levels, and dark green to blue indicates low concentrations.

To investigate the influence of liposomal encapsulation on the diffusion behavior of an active compound in a hydrogel-like environment, we employed a simplified ballistic gelatin model as a diffusion matrix and engaged in a 2D electron paramagnetic resonance imaging (EPRI) experiment (Figure 7). For the experiment, CBN liposomes were loaded with an aqueous solution of the hydrophilic spin probe 3-carbamoyl-PROXYL (3CP), Figure 7A. Comparative experiments were performed using both liposomal and free aqueous formulations to evaluate their relative mobility through the gelatin matrix. 

The EPRI results showed that after 30 min, the liposomal formulation exhibited significantly enhanced diffusion, with the 3CP signal detectable across the entire length of the gelatin plate. In contrast, the free 3CP solution penetrated only approximately 0.75 cm, indicating a markedly superior transport capacity of the liposome-encapsulated system within a hydrogel environment.

Although ballistic gelatin does not replicate the complex structure or lipid composition of the skin, it offers a simplified hydrogel-based matrix to assess relative diffusion behaviors. The significantly enhanced penetration of liposomal formulations compared to free drug solutions suggests that liposomes facilitate improved diffusion through semi-solid environments. This behavior is relevant for applications involving mucosal delivery, subcutaneous deposition, or soft-tissue targeting and supports the role of lipid-based carriers in enhancing distribution of actives. However, extrapolation to skin permeation requires further testing using biologically relevant models. Furthermore, this gelatin-based diffusion model offers a rapid and cost-effective platform for comparative screening of delivery systems during early-stage formulation development.

## 3. Materials and Methods

### 3.1. Chemicals and Reagents

The cannabinoid standards cannabidiol (CBD), cannabidiolic acid (CBDA), cannabigerol (CBG), and cannabinol (CBN) were purchased as high-purity (≥98%) isolates from Cayman Chemical (Ann Arbor, MI, USA). Identity and purity of each compound were confirmed by gas chromatography–mass spectrometry (GC–MS) and proton nuclear magnetic resonance (^1^H NMR) prior to analysis.

Cannabidiol diacetate (CBD-DOAc) was synthesized in-house from purified CBD isolate by acetylation of both phenolic hydroxyl groups. The final product was purified and characterized by GC–MS and ^1^H NMR to confirm structure and purity before use.

Acetic anhydride (≥99%), dichloromethane (DCM, ≥99.8%, anhydrous), triethylamine (TEA, ≥99%), and magnesium sulfate (MgSO_4_, anhydrous, ≥99.5%) used for the synthesis of CBD diacetate were obtained from Sigma-Aldrich (Merck, Darmstadt, Germany).

Deuterated chloroform (CDCl_3_, ≥99.8 atom % D) used for NMR spectroscopy was purchased from Sigma-Aldrich (Merck, Darmstadt, Germany).

Hydrogen peroxide, absolute ethanol, 2,2-diphenyl-1-picrylhydrazyl (DPPH), 5-doxyl stearic acid (5-DS), 3-carbamoyl-PROXYL (3CP), and ballistic gelatin used for EPR experiments were purchased from Sigma Aldrich (Merck, Darmstadt, Germany). 5-(Diethoxyphosphoryl)-5-methyl-1-pyrroline-N-oxide (DEPMPO) used for EPR spin-trapping experiments was purchased from Focus Biomolecules (Plymouth Meeting, PA, USA). Gas-permeable Teflon capillary tubes used for EPR sampling were obtained from Zeus Industries (Orangeburg, SC, USA).

All solvents were of analytical or HPLC grade and used without further purification unless otherwise specified.

### 3.2. Synthesis of CBD Diacetate

**Figure 8:**
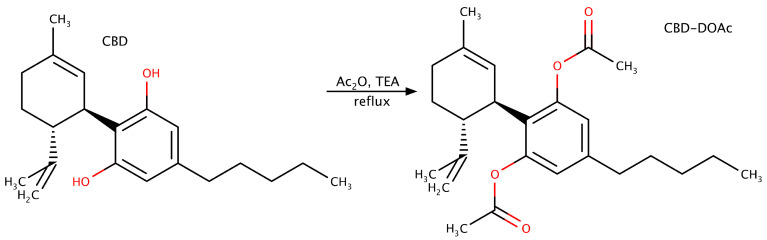
Synthesis of CBD diacetate.

The synthesis (Figure 8) was adapted from a previously reported method [33] with minor modifications, including the omission of 4-dimethylaminopyridine (DMAP) and the use of reflux in dichloromethane (DCM) for 10 h. A solution of cannabidiol (CBD, 15.8 g, 50 mm) in DCM (50 mL; 1.0 M) was treated with acetic anhydride (Ac_2_O, 10.4 mL, 110 mm, 2.2 equiv.) and triethylamine (Et_3_N, 13.9 mL, 100 mm, 2.0 equiv.). The reaction mixture was refluxed under an inert atmosphere for 10 h. Upon completion, as confirmed by GC–MS analysis, the mixture was quenched with saturated aqueous sodium bicarbonate (2 × 100 mL). The organic layer was separated, washed with brine (1 × 100 mL), dried over anhydrous magnesium sulfate (MgSO_4_), and filtered through a sintered glass funnel (DURAN^®^, porosity 2, 60 mm, DWK Life Sciences, Wertheim, Germany). The filtrate was concentrated under reduced pressure using a rotary evaporator (IKA RV 3 V, IKA, Staufen, Germany). The crude residue was purified by vacuum distillation using a Kugelrohr apparatus (Büchi Glass Oven B-585, Büchi, Flawil, Switzerland) to yield CBD diacetate (IUPAC name: [3-Acetyloxy-2-[(1R,6R)-6-isopropenyl-3-methylcyclohex-2-en-1-yl]-5-pentylphenyl] acetate) as a pale-yellow resin (13.92 g, 69.5% yield). The identity and purity of the product were confirmed by GC–MS and ^1^H NMR spectroscopy.

### 3.3. Sample Preparation

Each cannabinoid sample for GC–MS analysis was prepared by dissolving 10 mg of compound in 0.95 mL of acetone to obtain the primary stock solution. From this, 50 μL was further diluted in 0.95 mL of acetone to yield the final working solution. This two-step dilution ensured an appropriate analyte concentration for injection and optimized compound detection under the applied GC–MS conditions.

NMR samples were prepared by dissolving approximately 10 mg of each purified compound in 0.7 mL of deuterated chloroform (CDCl_3_). The resulting solutions were transferred to standard 5 mm NMR tubes and analyzed without further modification.

### 3.4. GC–MS Analysis

GC–MS analysis was carried out using an Agilent (Santa Clara, CA, USA) 6890N gas chromatograph coupled with a 5975B inert mass selective detector (MSD), equipped with an HP-5MS UI capillary column (30 m × 0.25 mm i.d., 0.25 µm film thickness; Agilent Technologies, Santa Clara, CA, USA). Helium (BP-grade) was used as the carrier gas at a constant flow rate of 1.2 mL/min. The injection port was maintained at 280 °C and operated in split mode (30:1). The oven temperature program was as follows: initial 60 °C (1 min hold), ramped at 15 °C/min to 300 °C, and held for 5 min (total run time: 22 min).

Sample injection (1 µL) was performed using an Agilent ALS autosampler. Electron impact (EI) ionization was applied at 70 eV with a mass scan range of *m*/*z* 24–550 and a solvent delay of 3 min. The transfer line and ion source temperatures were both maintained at 300 °C.

Data acquisition and analysis were performed using Agilent MSD ChemStation software (version G1701, Agilent, Santa Clara, CA, USA) with the Enhanced Data Analysis module.

Compound identification was performed by comparing the acquired mass spectra with entries in the NIST mass spectral database, authenticated reference standards, and published literature data. Additional confirmation was achieved using spectral data from the Wiley Registry of Mass Spectral Data, the SWGDRUG monographs, and GC–MS reference files provided by Cayman Chemical.

### 3.5. Nuclear Magnetic Resonance (NMR) Analysis

All NMR spectra were recorded on a Varian 400 MHz NMR spectrometer (Varian Inc., Palo Alto, CA, USA) at 399.74 MHz for ^1^H and 100.52 MHz for ^13^C, equipped with a 5 mm ATB probe head, standard pulse sequences, and deuterated chloroform (CDCl_3_) as solvent, at a temperature of 298 K. The spectra were referenced to the residual solvent signal (δH 7.24, δC 77.23).

### 3.6. Assessment of Radical Scavenging Activity Against DPPH Radicals

The potential of synthetic cannabinoids to neutralize DPPH free radicals was investigated using electron paramagnetic resonance (EPR) spectroscopy [50,51]. A 1 µL aliquot of the cannabinoid solution (1 mm in ethanol) was mixed with 29 µL of a freshly prepared 210 µm DPPH solution in ethanol. The reaction mixture was left to incubate for 2 min at room temperature, after which the EPR signal was immediately recorded. For control measurements, the same volume of pure ethanol was used in place of the cannabinoid solution.

EPR spectra were acquired using a Bruker ELEXSYS-II E540 EPR spectrometer (Bruker, Rheinstetten, Germany) operating in the X-band frequency range under the following instrumental settings: central magnetic field at 3500 G, microwave power of 10 mW, microwave frequency of 9.85 GHz, modulation frequency of 100 kHz, and modulation amplitude of 1 G.

The percentage of radical scavenging activity (RSA) was calculated using the following formula [52]:

$$RSA=\frac{\left(Ic-Ia\right)}{Ic}\times 100\%$$

where *Ic* represents the integrated EPR signal area of the control sample, and Ia corresponds to the signal of the sample containing the cannabinoid. Each measurement was performed in triplicate (*n* = 3).

### 3.7. Evaluation of Hydroxyl Radical Scavenging Capacity

To determine the ability of synthetic cannabinoids to quench hydroxyl radicals (•OH), a UV-induced radical generation system was employed in the presence of the spin-trapping agent DEPMPO [53,54]. A control mixture was prepared by combining 1 µL of DEPMPO (100 mm), 27 µL of deionized water, and 2 µL of diluted hydrogen peroxide solution (2.2 µL/mL). The solution was exposed to UV light for 30 s to generate hydroxyl radicals, and the EPR spectrum was recorded after a 5 min interval. To assess radical scavenging, 1 µL of the cannabinoid solution (1 mm in ethanol) was added in place of distilled water, and the same irradiation and recording protocol was followed. EPR acquisition parameters matched those described in the DPPH assay. Each measurement was performed in triplicate (*n* = 3).

### 3.8. Determination of Superoxide Anion Scavenging Activity

The scavenging activity of synthetic cannabinoids toward superoxide anion radicals (O_2_•^−^) was analyzed using a DEPMPO-based spin trapping method [54,55]. A reaction mixture was prepared by combining 18 µL of deionized water, 10 µL of hydrogen peroxide (10 M), and 1 µL of DEPMPO (100 mm). Subsequently, 1 µL of the cannabinoid solution (1 mm in ethanol) was added. Radical generation was initiated by UV irradiation for 30 s, followed by a 2 min incubation.

The reaction mixture was then loaded into a gas-permeable Teflon tube for EPR measurement. A control experiment was performed by substituting the cannabinoid solution with ethanol. All spectra were recorded under the same conditions as described for the DPPH assay. Each measurement was performed in triplicate (*n* = 3).

### 3.9. Preparation of CBN-Loaded Soy Lecithin Liposomes

Liposomes were formulated using a modified thin-film hydration technique [55,56], with CBN as the active compound and soy lecithin as the phospholipid matrix. Briefly, CBN (5% *w*/*w*) and soy lecithin (95% *w*/*w*) were co-dissolved in a 4:1 (*v*/*v*) mixture of chloroform and methanol in a round-bottom flask. The organic solvents were slowly evaporated at room temperature using a rotary vacuum evaporator (IKA RV3 V, IKA, Staufen, Germany), leaving a thin lipid film on the inner wall of the flask.

The resulting dry film was hydrated with 2 mL of ultrapure water (18 MΩ·cm) in successive additions of 250 µL. After each addition, the suspension was vortexed (IKA Vortex 3, IKA, Staufen, Germany) for 3 min and then subjected to ultrasound treatment for 3 min using a cooled ultrasonic water bath (ACP-120H, MRC lab, Holon, Israel). To further standardize the liposome size distribution, the liposomal dispersion underwent additional sonication for 20 min to promote vesicle formation and size reduction. This was performed using an ultrasound probe (LHDM502, Colo LabExperts equipment, Novo Mesto, Slovenia) set to 15% of power, during 15 min in a 20 s on/20 s off regime. A parallel batch of liposomes containing only soy lecithin (100%) was prepared following the same protocol and served as a control.

### 3.10. Size and Zeta Potential Characterization of Liposomes

The hydrodynamic diameter of the CBN-loaded and control soy lecithin liposomes was measured using dynamic light scattering (DLS) [57]. In this method, laser light is directed at particles undergoing Brownian motion, and the resulting scattered light fluctuations are recorded at a fixed angle over time. These intensity fluctuations are used to generate a correlation function, which provides information on the translational diffusion of the particles. Because the diffusion rate is inversely proportional to particle size, the analysis allows for calculation of the size distribution within the sample.

For each measurement, 100 µL of the liposome suspension (either CBN-loaded or blank) was diluted to 1 mL with deionized water. Measurements were performed at 25 °C [44] using the Malvern Zetasizer Nano ZS (Malvern Instruments, Worcestershire, UK) DLS device. Each measurement was performed in triplicate (*n* = 3).

### 3.11. Spin Labeling of Liposomes for EPR Analysis

To investigate the dynamic properties of the lipid bilayer, spin-labeling experiments were conducted using 5-doxyl stearic acid (5-DS), a nitroxide-labeled fatty acid that integrates into lipid membranes near the bilayer surface [58]. The motion of the doxyl radical within the membrane environment affects the shape of the EPR spectrum, allowing insight into the local membrane fluidity and order [55].

A 7.5 µL aliquot of 1 mm 5-DS in ethanol was placed in a 500 µL centrifugal test tube and evaporated under vacuum (Concentrator Plus, Eppendorf, Hamburg, Germany) for 5 min to form a thin spin-probe film. Subsequently, 30 µL of the CBN-containing or control soy lecithin liposome suspension was added. The mixture was vortexed for 2 min (IKA Vortex 3, IKA, Staufen, Germany) and incubated at room temperature for 5 min to facilitate incorporation of the spin label into the lipid bilayer.

The spin-labeled liposome suspension was transferred into a gas-permeable Teflon tube (1 mm internal diameter) for EPR measurement. Spectra were recorded under the following conditions: center magnetic field 3500 G, microwave power 10 mW, microwave frequency 9.85 GHz, modulation frequency 100 kHz, and modulation amplitude 1 G. Each measurement was performed in triplicate (*n* = 3).

### 3.12. Evaluation of Radical Scavenging Activity of Liposomes

The antioxidant potential of CBN-loaded liposomes was evaluated against DPPH, hydroxyl (•OH), and superoxide anion (O_2_•^−^) radicals using the same experimental conditions and protocols previously described for the free compound. In each assay, the liposomal suspension was applied in an equivalent volume (1 µL) as in the tests with non-encapsulated CBN, allowing for direct comparison of radical scavenging efficacy between the free and liposome-encapsulated forms. Each measurement was performed in triplicate (*n* = 3).

### 3.13. Experimental Design for Investigating Liposomal Mobility in a Semi-Solid Environment

To enable EPR imaging analysis, CBN-loaded liposomes were prepared using a modified thin-film hydration method, in which the standard aqueous phase was replaced with a 15 mm solution of the hydrophilic spin probe 3-carbamoyl-PROXYL (3CP). As a control, an aqueous 3CP solution of the same concentration was prepared in distilled water.

A ballistic gelatin matrix was employed as a simplified hydrogel model to assess the diffusion behavior of the test samples. Gelatin plates were cast with dimensions of 5 mm (width) × 17 mm (length) × 2 mm (thickness). For each sample, either 10 μL of the 3CP solution or the liposomal formulation was applied to the top edge of a separate gelatin plate (Figure 7). Plates were maintained in a vertical orientation at room temperature (~25 °C) throughout the experiment. The penetration distances were determined directly from the 2D images using the scale bar.

The spatial distribution of the 3CP spin probe within the gelatin matrix was monitored using a 2D electron paramagnetic resonance imaging (EPRI) experiment in the XY-plane (Figure 7). Measurements were conducted on a Bruker ELEXSYS-II E540 EPR spectrometer (Bruker, Rheinstetten, Germany) equipped with magnetic field gradient coils and operating in the X-band frequency range. The following parameters were used: microwave power 10 mW, microwave frequency 9.8 GHz, modulation frequency 100 kHz, modulation amplitude 2 G, and magnetic field gradient 20 G·cm^−1^. Images were acquired 30 min after sample application to assess the extent of 3CP diffusion through the gelatin matrix. Data analysis was performed using Xepr software, version 2.6b.84 (Bruker BioSpin, Rheinstetten, Germany).

### 3.14. Statistical Analysis

Unless otherwise stated, all experiments were performed in triplicate (*n* = 3), and results are expressed as mean ± standard deviation (SD). Statistical analyses were conducted using Statistica 12.0 64-bit Statistical Software (StatSoft (Europe) GmbH, Hamburg, Germany). Differences between groups were evaluated using one-way analysis of variance (ANOVA) followed by Tukey’s post-hoc test. A value of *p* < 0.05 was considered statistically significant.

## 4. Conclusions

This study highlights the multifunctional potential of synthetic cannabinoids, particularly cannabinol (CBN), in free and liposome-encapsulated forms. Among the five investigated compounds, CBN consistently exhibited the highest radical scavenging activity across all tested reactive species, demonstrating a balanced and potent antioxidant profile. Based on these results, CBN was selected for formulation into soy lecithin liposomes. The resulting CBN-loaded liposomes were nanosized, stable, and exhibited good colloidal properties. Dynamic light scattering confirmed their average diameter around 106 nm, while spin-labeling EPR experiments revealed an increase in membrane order upon CBN incorporation, suggesting enhanced bilayer stability without compromising fluidity. Importantly, CBN retained significant antioxidant capacity even after encapsulation, supporting the use of liposomes as effective carriers for hydrophobic bioactive molecules. Although a moderate reduction in radical scavenging efficiency was observed compared to the free compound, the encapsulated form remained functionally active against DPPH, hydroxyl, and superoxide radicals. Complementary EPR imaging studies further demonstrated significantly enhanced penetration of liposomal formulations compared to free drug solutions through semi-solid environments, suggesting that liposomes facilitate improved diffusion. This model proved to be a rapid and cost-effective tool for comparative assessment of formulation mobility during early development stages. While the findings support the suitability of lipid-based carriers for mucosal, subcutaneous, or soft-tissue applications, their relevance to skin permeation remains to be validated through studies in biologically representative skin models. Future research should therefore include in vitro and ex vivo skin permeation experiments, long-term stability testing under different storage conditions, and in vivo evaluations of safety and efficacy. Potential applications extend to antioxidant-enriched dermal formulations aimed at mitigating oxidative stress-related skin conditions, such as photoaging, chronic inflammation, or impaired wound healing, as well as to other mucosal delivery systems for hydrophobic antioxidants. Limitations of this study include the absence of biological activity testing in cell or tissue models, potential variability in liposomal stability under physiological conditions, and the need to assess large-scale manufacturing feasibility. Addressing these aspects will be essential for translating CBN-loaded liposomes from a promising laboratory formulation into a clinically relevant dermal antioxidant delivery platform.

## Figures and Tables

**Table 1:** GC–MS retention times and TIC purity of cannabinoid standards.

Compound	RT (min)	Area % TIC	Other Peaks > 0.5% TIC?	Purity Reported
CBN	16.364	100	No	>99%
CBD-DOAc	15.832	100	No	>99%
CBDA ^1^	15.458	100	No	>99%
CBG	16.236	100	No	>99%
CBD	15.458	100	No	>99%

^1^ Each standard was analyzed individually. The CBDA sample showed a mass spectrum identical to CBD, consistent with thermal decarboxylation. The identity of CBDA was proven by NMR (see Section 2.2).

## Data Availability

Essential data are contained within the article and Supplementary Materials. Additionally, the dataset generated and analyzed during this study, which contributed to the article, is available from the corresponding authors upon reasonable request, provided that such requests do not compromise intellectual property interests.

## Supplementary Materials

The following supporting information can be downloaded at https://www.mdpi.com/article/10.3390/molecules30163433/s1: Figure S1: GC–MS analysis of CBN. Figure S2: ^1^H NMR spectrum of CBN. Figure S3: ^13^C NMR spectrum of CBN. Figure S4: GC–MS analysis of CBD diacetate. Figure S5: ^1^H NMR spectrum of CBD diacetate. Figure S6: ^13^C NMR spectrum of CBD diacetate. Figure S7: GC–MS analysis of CBDA. Figure S8: ^1^H NMR spectrum of CBDA. Figure S9: ^13^C NMR spectrum of CBDA. Figure S10: GC–MS analysis of CBG. Figure S11: ^1^H NMR spectrum of CBG. Figure S12: ^13^C NMR spectrum of CBG. Figure S13: GC–MS analysis of CBD. Figure S14: ^1^H NMR spectrum of CBD. Figure S15: ^13^C NMR spectrum of CBD. Table S1: Comparative antiradical activities of pure CBN and CBN-loaded liposomes. Refs. [59,60,61,62,63] are cited in the Supplementary Materials.

## Abbreviations

The following abbreviations are used in this manuscript:

^1^H NMR	Proton Nuclear Magnetic Resonance
^13^C NMR	Carbon-13 Nuclear Magnetic Resonance
3CP	3-Carbamoyl-PROXYL (hydrophilic spin probe)
CBD	Cannabidiol
CBD-DOAc	Cannabidiol Diacetate
CBDA	Cannabidiolic Acid
CBG	Cannabigerol
CBN	Cannabinol
CDCl_3_	Deuterated Chloroform
DCM	Dichloromethane
DEPMPO	5-(Diethoxyphosphoryl)-5-methyl-1-pyrroline N-oxide (spin trapping agent)
DLS	Dynamic Light Scattering
DMAP	4-Dimethylaminopyridine
DPPH	2,2-Diphenyl-1-picrylhydrazyl
EPR	Electron Paramagnetic Resonance
EPRI	Electron Paramagnetic Resonance Imaging
Et_3_N	Triethylamine
GC–MS	Gas Chromatography–Mass Spectrometry
IL	Interleukin
log P	Octanol–Water Partition Coefficient (indicator of lipophilicity)
MS	Mass Spectrometry
NIST	National Institute of Standards and Technology
NMR	Nuclear Magnetic Resonance
O_2_•^−^	Superoxide Anion Radical
ROS	Reactive Oxygen Species
RT	Retention Time
SD	Standard Deviation
SWGDRUG	Scientific Working Group for the Analysis of Seized Drugs
THC	Δ^9^-Tetrahydrocannabinol
TIC	Total Ion Chromatogram
UV	Ultraviolet

## References

[1] Farhan M. (2024). The Promising Role of Polyphenols in Skin Disorders. Molecules.

[2] Bhat A.H., Dar K.B., Anees S., Zargar M.A., Masood A., Sofi M.A., Ganie S.A. (2015). Oxidative Stress, Mitochondrial Dysfunction and Neurodegenerative Diseases; a Mechanistic Insight. Biomed. Pharmacother..

[3] He X., Wan F., Su W., Xie W. (2023). Research Progress on Skin Aging and Active Ingredients. Molecules.

[4] Hallan S.S., Ferrara F., Cortesi R., Sguizzato M. (2025). Potential of the Nano-Encapsulation of Antioxidant Molecules in Wound Healing Applications: An Innovative Strategy to Enhance the Bio-Profile. Molecules.

[5] Ndhlala A.R., Moyo M., Van Staden J. (2010). Natural Antioxidants: Fascinating or Mythical Biomolecules?. Molecules.

[6] Charlton N.C., Mastyugin M., Török B., Török M. (2023). Structural Features of Small Molecule Antioxidants and Strategic Modifications to Improve Potential Bioactivity. Molecules.

[7] Rajendran P., Nandakumar N., Rengarajan T., Palaniswami R., Gnanadhas E.N., Lakshminarasaiah U., Gopas J., Nishigaki I. (2014). Antioxidants and Human Diseases. Clin. Chim. Acta.

[8] Jardim F.R., De Rossi F.T., Nascimento M.X., Da Silva Barros R.G., Borges P.A., Prescilio I.C., De Oliveira M.R. (2018). Resveratrol and Brain Mitochondria: A Review. Mol. Neurobiol..

[9] Go Y.K., Leal C. (2021). Polymer–Lipid Hybrid Materials. Chem. Rev..

[10] Jangde R., Elhassan G.O., Khute S., Singh D., Singh M., Sahu R.K., Khan J. (2022). Hesperidin-Loaded Lipid Polymer Hybrid Nanoparticles for Topical Delivery of Bioactive Drugs. Pharmaceuticals.

[11] Zagórska-Dziok M., Ziemlewska A., Bujak T., Nizioł-Łukaszewska Z., Hordyjewicz-Baran Z. (2021). Cosmetic and Dermatological Properties of Selected Ayurvedic Plant Extracts. Molecules.

[12] Duczmal D., Bazan-Wozniak A., Niedzielska K., Pietrzak R. (2024). Cannabinoids—Multifunctional Compounds, Applications and Challenges—Mini Review. Molecules.

[13] Atalay S., Jarocka-Karpowicz I., Skrzydlewska E. (2019). Antioxidative and Anti-Inflammatory Properties of Cannabidiol. Antioxidants.

[14] Hampson A.J., Grimaldi M., Axelrod J., Wink D. (1998). Cannabidiol and (−)Δ^9^-Tetrahydrocannabinol Are Neuroprotective Antioxidants. Proc. Natl. Acad. Sci. USA.

[15] Brierley D.I., Samuels J., Duncan M., Whalley B.J., Williams C.M. (2016). Cannabigerol Is a Novel, Well-Tolerated Appetite Stimulant in Pre-Satiated Rats. Psychopharmacology.

[16] Dawidowicz A.L., Olszowy-Tomczyk M., Typek R. (2021). CBG, CBD, Δ^9^-THC, CBN, CBGA, CBDA and Δ^9^-THCA as Antioxidant Agents and Their Intervention Abilities in Antioxidant Action. Fitoterapia.

[17] Wilkinson J.D., Williamson E.M. (2007). Cannabinoids Inhibit Human Keratinocyte Proliferation through a Non-CB1/CB2 Mechanism and Have a Potential Therapeutic Value in the Treatment of Psoriasis. J. Dermatol. Sci..

[18] Wang X., Zhang H., Liu Y., Xu Y., Yang B., Li H., Chen L. (2023). An Overview on Synthetic and Biological Activities of Cannabidiol (CBD) and Its Derivatives. Bioorg. Chem..

[19] Mahmoudinoodezh H., Telukutla S.R., Bhangu S.K., Bachari A., Cavalieri F., Mantri N. (2022). The Transdermal Delivery of Therapeutic Cannabinoids. Pharmaceutics.

[20] Ferreira B.P., Costa G., Mascarenhas-Melo F., Pires P.C., Heidarizadeh F., Giram P.S., Mazzola P.G., Cabral C., Veiga F., Paiva-Santos A.C. (2023). Skin Applications of Cannabidiol: Sources, Effects, Delivery Systems, Marketed Formulations and Safety. Phytochem. Rev..

[21] Filipiuc S.-I., Neagu A.-N., Uritu C.M., Tamba B.-I., Filipiuc L.-E., Tudorancea I.M., Boca A.N., Hâncu M.F., Porumb V., Bild W. (2023). The Skin and Natural Cannabinoids–Topical and Transdermal Applications. Pharmaceuticals.

[22] Lefebvre È., Tawil N., Yahia L. (2024). Transdermal Delivery of Cannabidiol for the Management of Acute Inflammatory Pain: A Comprehensive Review of the Literature. Int. J. Mol. Sci..

[23] Calienni M.N., Scavone M.A., Sanguinetti A.P., Corleto M., Di Meglio M.R., Raies P., Cristos D.S., Maffia P.C., Montanari J. (2024). Lipid Nanoparticle Formulations for the Skin Delivery of Cannabidiol. Pharmaceutics.

[24] Musielak E., Krajka-Kuźniak V. (2024). Liposomes and Ethosomes: Comparative Potential in Enhancing Skin Permeability for Therapeutic and Cosmetic Applications. Cosmetics.

[25] Holt A.K., Poklis J.L., Peace M.R. (2022). Δ^8^-THC, THC-O-Acetates and CBD-di-O-Acetate: Emerging Synthetic Cannabinoids Found in Commercially Sold Plant Material and Gummy Edibles. J. Anal. Toxicol..

[26] Munger K., Jensen R., Strongin R.M. (2022). Vaping Cannabinoid Acetates Leads to Ketene Formation. Chem. Res. Toxicol..

[27] Dussy F.E., Hamberg C., Luginbühl M., Schwerzmann T., Briellmann T.A. (2005). Isolation of Delta9-THCA-A from Hemp and Analytical Aspects Concerning the Determination of Delta9-THC in Cannabis Products. Forensic Sci. Int..

[28] Franzin M., Di Lenardo R., Ruoso R., Addobbati R. (2025). Incomplete Decarboxylation of Acidic Cannabinoids in GC–MS Leads to Underestimation of the Total Cannabinoid Content in Cannabis Oils Without Derivatization. Pharmaceutics.

[29] Seo C., Jeong M., Lee S., Kim E.J., Rho S., Cho M., Lee Y.S., Hong J. (2022). Thermal Decarboxylation of Acidic Cannabinoids in Cannabis Species: Identification of Transformed Cannabinoids by UHPLC-Q/TOF–MS. J. Anal. Sci. Technol..

[30] Choi Y.H., Hazekamp A., Peltenburg-Looman A.M.G., Frédérich M., Erkelens C., Lefeber A.W.M., Verpoorte R. (2004). NMR Assignments of the Major Cannabinoids and Cannabiflavonoids Isolated from Flowers of *Cannabis sativa*. Phytochem. Anal..

[31] Hazekamp A., Choi Y.H., Verpoorte R. (2004). Quantitative Analysis of Cannabinoids from *Cannabis sativa* Using ^1H NMR. Chem. Pharm. Bull..

[32] Barthlott I., Scharinger A., Golombek P., Kuballa T., Lachenmeier D.W. (2021). A Quantitative ^1^H NMR Method for Screening Cannabinoids in CBD Oils. Toxics.

[33] Fiorito D., Tessaro D., Sangalli F., Nobbio C., Nebuloni M., Vezzini M., Brenna E., Parmeggiani F. (2024). Valorisation of the Industrial Hemp Residue from Essential Oil Production by Recovery of Cannabidiol and Chemo Enzymatic Conversion to Cannabielsoin. Green Chem..

[34] Pellati F., Brighenti V., Sperlea J., Marchetti L., Bertelli D., Benvenuti S. (2020). (–)-Cannabidiolic Acid, a Still Overlooked Bioactive Compound—An Introductory Review and Preliminary Research. Molecules.

[35] Colella M.F., Salvino R.A., Gaglianò M., Litrenta F., Oliviero Rossi C., Le Pera A., De Luca G. (2022). NMR Spectroscopy Applied to the Metabolic Analysis of Natural Extracts of *Cannabis sativa*. Molecules.

[36] Buczek A., Rzepiela K., Kupka T., Broda M.A. (2025). Impact of OH•••π Hydrogen Bond on IR and NMR Parameters of Cannabidiol: Theoretical and Experimental Study. Molecules.

[37] Codd L.S., Durrant S.J. (2008). Magnetic Resonance Microscopy: Spatially Resolved NMR Techniques and Applications.

[38] Berliner L.J. (1976). Spin Labeling Theory and Applications.

[39] Cevc G. (2004). Lipid Vesicles and Other Colloids as Drug Carriers on the Skin. Adv. Drug Deliv. Rev..

[40] Nasab M.E., Takzaree N., Mirzaee Saffari P., Partoazar A. (2019). In vitro antioxidant activity and in vivo wound-healing effect of lecithin liposomes: A comparative study. J. Comp. Eff. Res..

[41] Chen Y., Wu Q., Zhang Z., Yuan L., Liu X., Zhou L. (2012). Preparation of Curcumin-Loaded Liposomes and Evaluation of Their Skin Permeation and Pharmacodynamics. Molecules.

[42] Ricci A., Stefanuto L., Gasperi T., Bruni F., Tofani D. (2024). Lipid Nanovesicles for Antioxidant Delivery in Skin: Liposomes, Ufasomes, Ethosomes, and Niosomes. Antioxidants.

[43] Liu C., Cheng X., Wu Y., Xu W., Xia H., Jia R., Liu Y., Shen S., Xu Y., Cheng Z. (2023). Antioxidant Activity of Quercetin-Containing Liposomes-in-Gel and Its Effect on Prevention and Treatment of Cutaneous Eczema. Pharmaceutics.

[44] Prevete G., Simonis B., Mazzonna M., Mariani F., Donati E., Sennato S., Ceccacci F., Bombelli C. (2023). Resveratrol and Resveratrol-Loaded Galactosylated Liposomes: Anti-Adherence and Cell Wall Damage Effects on Staphylococcus aureus and MRSA. Biomolecules.

[45] Kasprzak-Drozd K., Niziński P., Hawrył A., Gancarz M., Hawrył D., Oliwa W., Pałka M., Markowska J., Oniszczuk A. (2024). Potential of Curcumin in the Management of Skin Diseases. Int. J. Mol. Sci..

[46] Franzè S., Angelo L., Casiraghi A., Minghetti P., Cilurzo F. (2022). Design of Liposomal Lidocaine/Cannabidiol Fixed Combinations for Local Neuropathic Pain Treatment. Pharmaceutics.

[47] Lapteva M., Faro Barros J., Kalia Y.N. (2024). Cutaneous Delivery and Biodistribution of Cannabidiol in Human Skin after Topical Application of Colloidal Formulations. Pharmaceutics.

[48] Lodzki M., Godin B., Rakou L., Mechoulam R., Gallily R., Touitou E. (2003). Cannabidiol—Transdermal Delivery and Anti-Inflammatory Effect in a Murine Model. J. Control. Release.

[49] Zapata K., Rosales S., Rios A., Rojano B., Toro-Mendoza J., Riazi M., Franco C.A., Cortés F.B. (2023). Nanoliposomes for Controlled Release of Cannabinodiol at Relevant Gastrointestinal Conditions. ACS Omega.

[50] Nakarada Đ., Marković S., Popović M., Dimitrijević M., Rakić A., Mojović M. (2021). Redox Properties of Grape Wine Skin Extracts from the Šumadija Region: An Electron Paramagnetic Resonance Study. Hosp. Pharmacol. Int. Multidiscip. J..

[51] Sanna D., Delogu G., Mulas M., Schirra M., Fadda A. (2012). Determination of Free Radical Scavenging Activity of Plant Extracts Through DPPH Assay: An EPR and UV-Vis Study. Food Anal. Methods.

[52] Savić J., Nakarada Đ., Stupar S., Tubić L., Milutinović M., Mojović M., Devrnja N. (2024). Glutathione Involvement in Potato Response to French Marigold Volatile Organic Compounds. Antioxidants.

[53] Dikalov S., Kirilyuk I., Grigor’ev I. (1996). Spin Trapping of O-, C-, and S-Centered Radicals and Peroxynitrite by 2H-Imidazole-1-Oxides. Biochem. Biophys. Res. Commun..

[54] Glavinić U., Nakarada Đ., Stevanović J., Gašić U., Ristanić M., Mojović M., Stanimirović Z. (2025). Chemical Composition and Antioxidant Activity of Prokupac Grape Pomace Extract: Implications for Redox Modulation in Honey Bee Cells. Antioxidants.

[55] Nakarada Đ., Pejin B., Tommonaro G., Mojović M. (2020). Liposomal Integration Method for Assessing Antioxidative Activity of Water Insoluble Compounds Towards Biologically Relevant Free Radicals: Example of Avarol. J. Liposome Res..

[56] Stojković T., Mitrović A., Kastratović D., Nakarada Đ., Mojović M. (2023). EPRI Approach on Studying Deep Skin Penetration of Tretinoin-Containing Liposomes. Hosp. Pharmacol. Int. Multidiscip. J..

[57] Berne B.J., Pecora R. (1976). Dynamic Light Scattering with Applications to Chemistry, Biology and Physics.

[58] Yuann J.M.P., Morse R. (1999). Determination by Photoreduction of Flip-Flop Kinetics of Spin-Labeled Stearic Acids across Phospholipid Bilayers. Biochim. Biophys. Acta Biomembr..

[59] National Institute of Standards and Technology (NIST) NIST Chemistry WebBook, SRD 69; Gaithersburg, MD, USA. https://webbook.nist.gov/cgi/cbook.cgi?ID=C521357&Mask=200#Mass-Spec.

[60] Cayman Chemical GC–MS Spectral Data Sheet for Cannabidiol Diacetate (CAS 58452-86-9). https://cdn.caymanchem.com/cdn/gcms/36424-0646266-GCMS.pdf.

[61] PubChem Cannabidiolic Acid (CBDA); CID: 6440999. Spectral Information (Wiley Registry of Mass Spectral Data). https://pubchem.ncbi.nlm.nih.gov/compound/Cannabidiolic-acid#section=Spectral-Information.

[62] Scientific Working Group for the Analysis of Seized Drugs (SWGDRUG) Monograph: Cannabigerol (CBG). https://www.swgdrug.org/Monographs/Cannabigerol.pdf.

[63] National Institute of Standards and Technology (NIST) NIST Chemistry WebBook, SRD 69; Gaithersburg, MD, USA. Cannabidiol (CAS 13956-29-1). https://webbook.nist.gov/cgi/cbook.cgi?ID=C13956291&Mask=200#Mass-Spec.

