# Supplementary material for: From Analytical Profiling to Liposomal Delivery: Cannabinol as a Model for Antioxidant Encapsulation and Diffusion Enhancement
Source: Molecules. 2025 Aug 20;30(16):3433. doi: 10.3390/molecules30163433 (PMC12388803; doi:10.3390/molecules30163433)
Supplement: Supplementary file 1 [file molecules-30-03433-s001.zip › molecules-3813069-supplementary.pdf]

-Supplementary Material-

Table of Contents

Figure S1. GC–MS analysis of CBN 2
Figure S2. ^{1}H NMR spectrum of CBN 3
Figure S3. ^{13}C NMR spectrum of CBN 3
Figure S4. GC–MS analysis of CBD diacetate 4
Figure S5. ^{1}H NMR spectrum of CBD diacetate 5
Figure S6. ^{13}C NMR spectrum of CBD diacetate 5
Figure S7. GC–MS analysis of CBDA 6
Figure S8. ^{1}H NMR spectrum of CBDA 7
Figure S9. ^{13}C NMR spectrum of CBDA 7
Figure S10. GC–MS analysis of CBG 8
Figure S11. ^{1}H NMR spectrum of CBG 9
Figure S12. ^{13}C NMR spectrum of CBG 9
Figure S13. GC–MS analysis of CBD 10
Figure S14. ^{1}H NMR spectrum of CBD 11
Figure S15. ^{13}C NMR spectrum of CBD 11
Table S1. Comparative antiradical activities of pure CBN and CBN-loaded liposomes 12

References 12

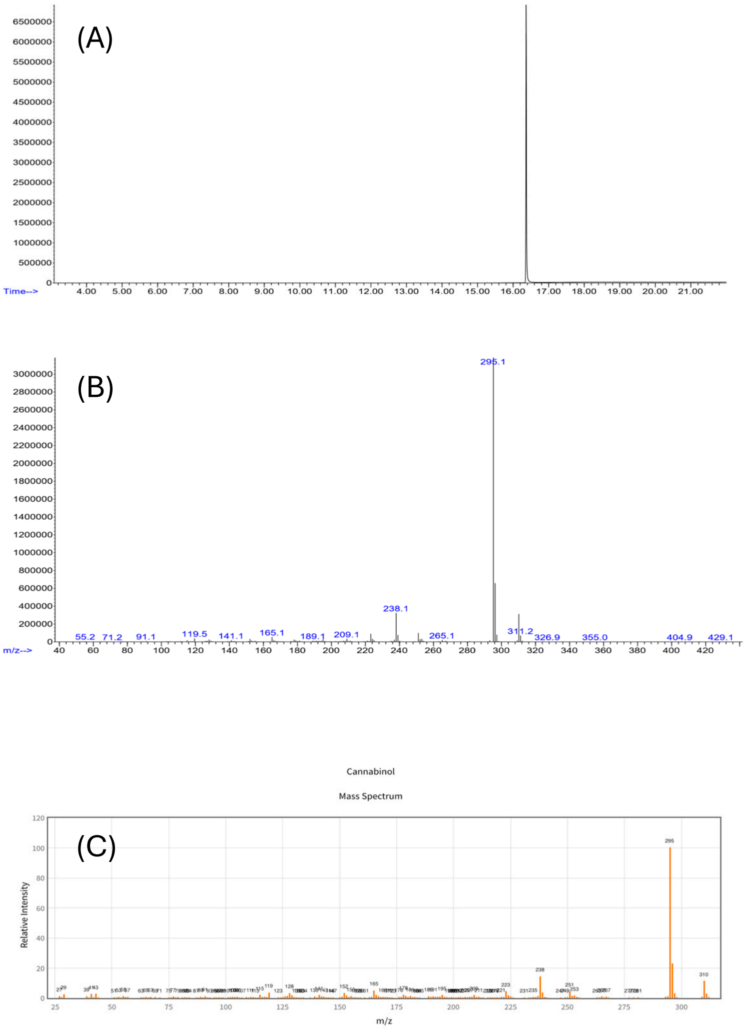

Figure S1. GC–MS analysis of CBN

(**A**) Total ion chromatogram (TIC) of the CBN sample analyzed in this study.

(**B**) Experimental electron ionization (EI) mass spectrum (70 eV) corresponding to the peak at RT = 16,370 min.

(**C**) Reference EI mass spectrum of CBN obtained from the NIST Chemistry WebBook [NIST1].

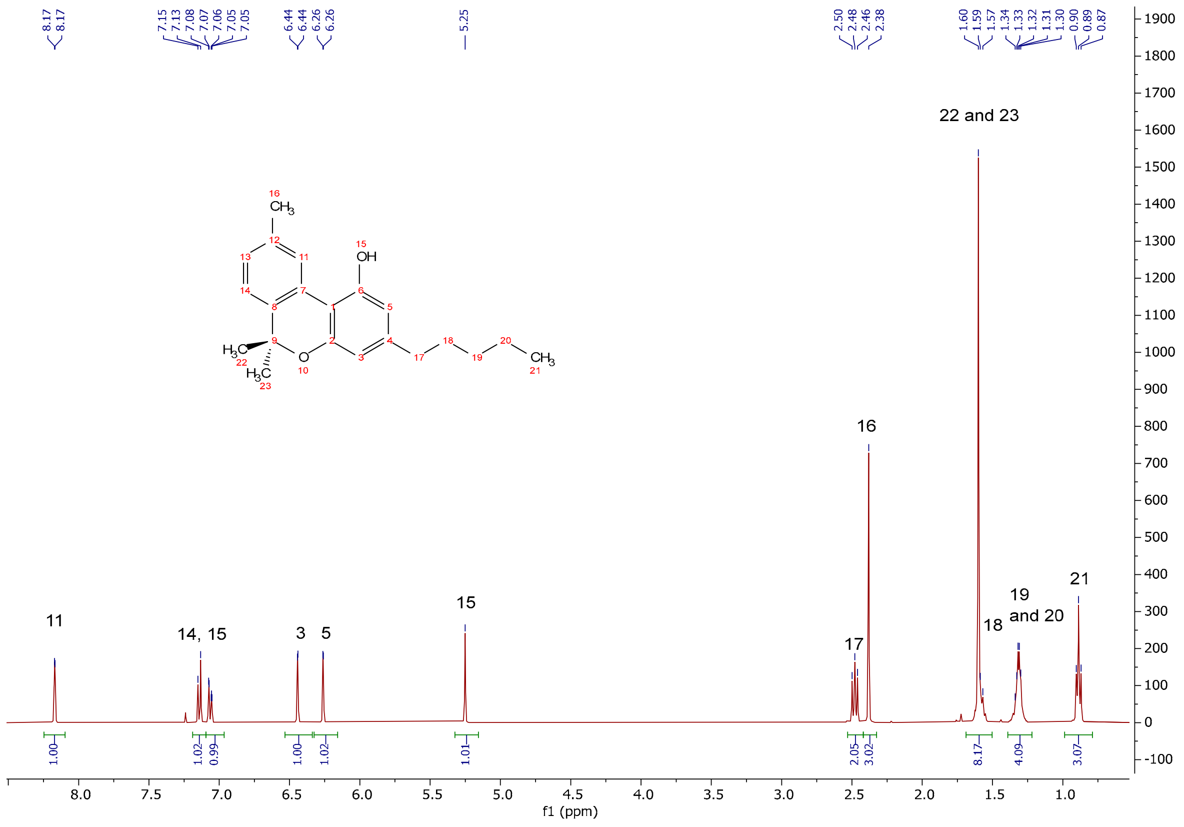

Figure S2. ^{1}H NMR spectrum of CBN

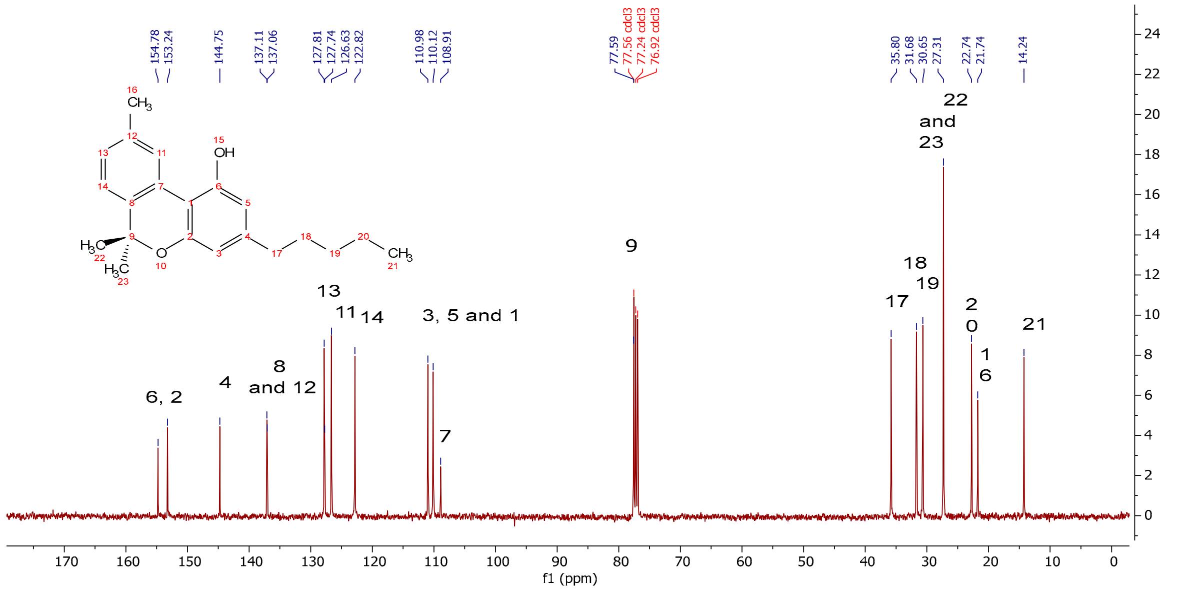

Figure S3. ^{13}C NMR spectrum of CBN

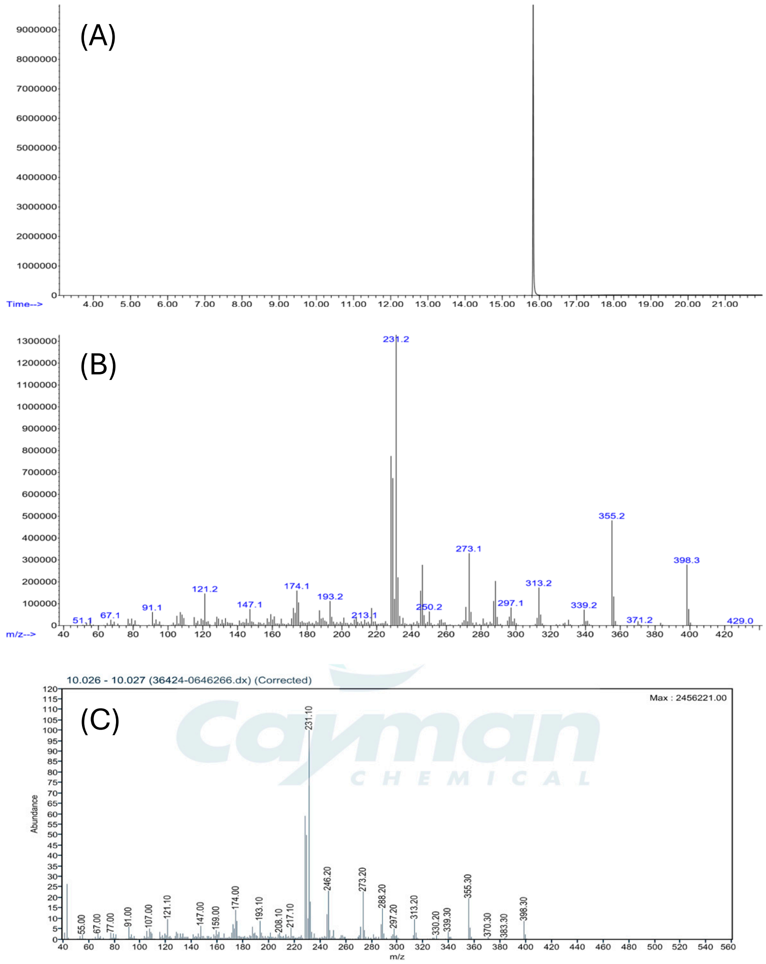

Figure S4. GC–MS analysis of CBD diacetate

(**A**) Total ion chromatogram (TIC) of the CBD-DOAc sample analyzed in this study.

(**B**) Experimental electron ionization (EI) mass spectrum (70 eV) corresponding to the peak at RT = 15,832 min.

(**C**) Reference EI mass spectrum of CBD-DOAc obtained from Cayman Chemical GC–MS spectral library [Cayman2].

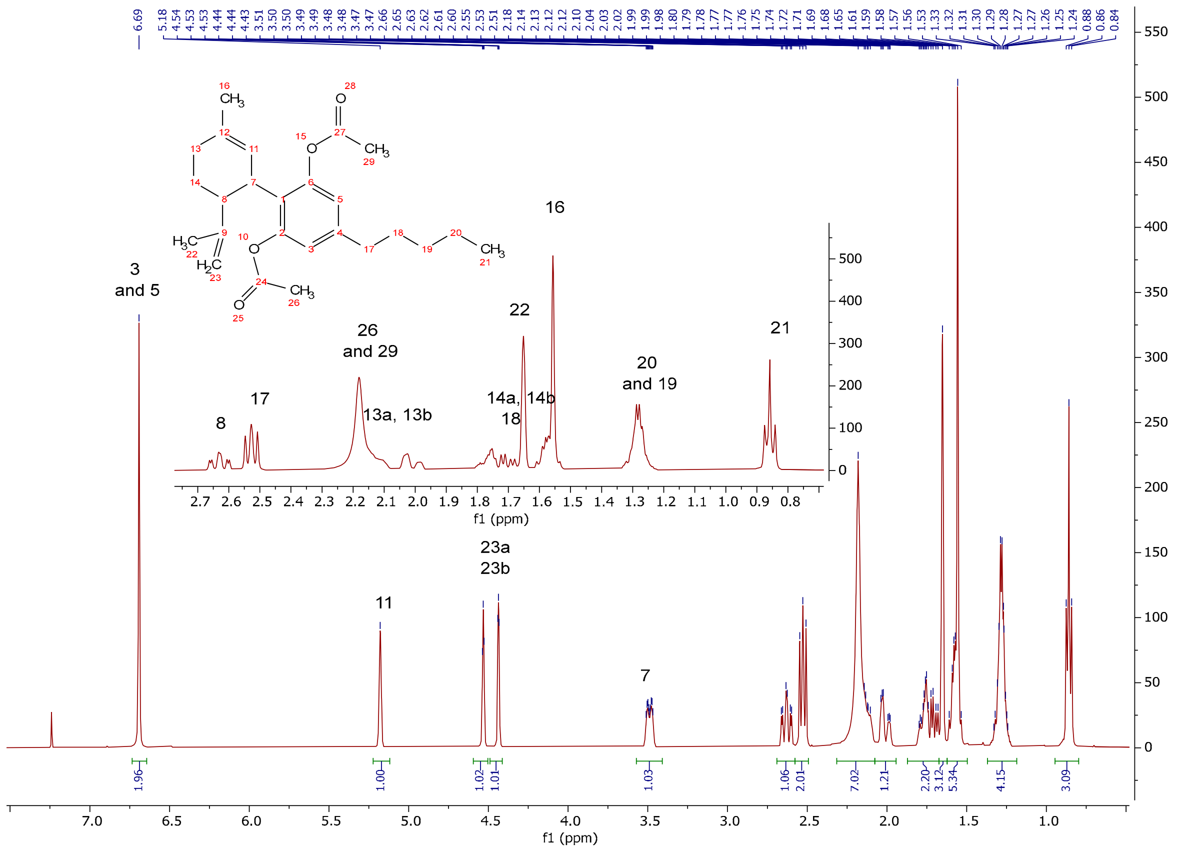

Figure S5. ^{1}H NMR spectrum of CBD diacetate

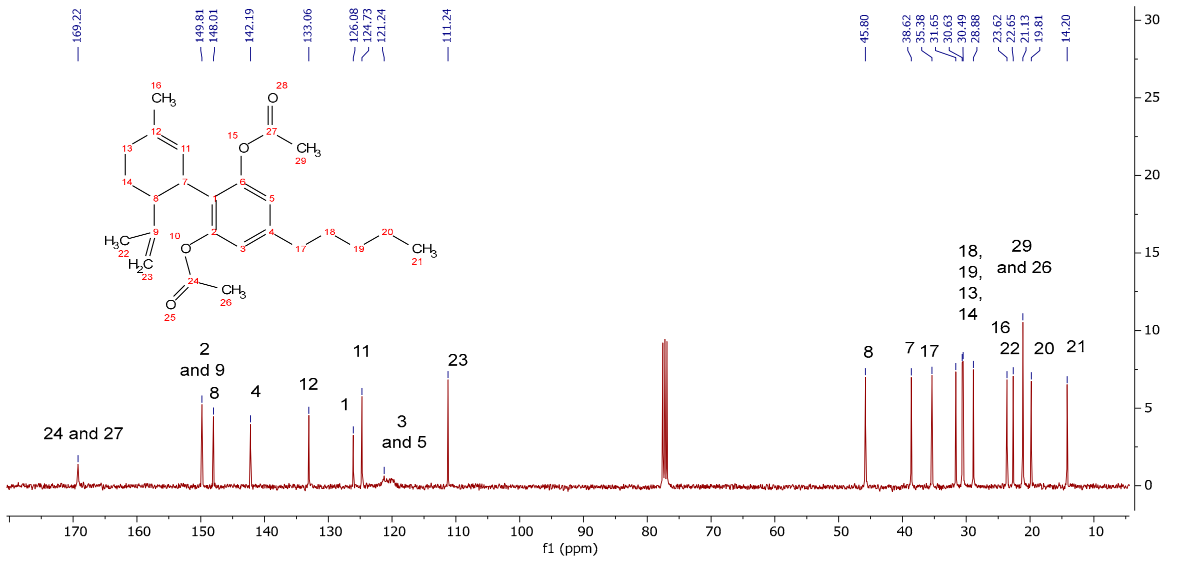

Figure S6. ^{13}C NMR spectrum of CBD diacetate

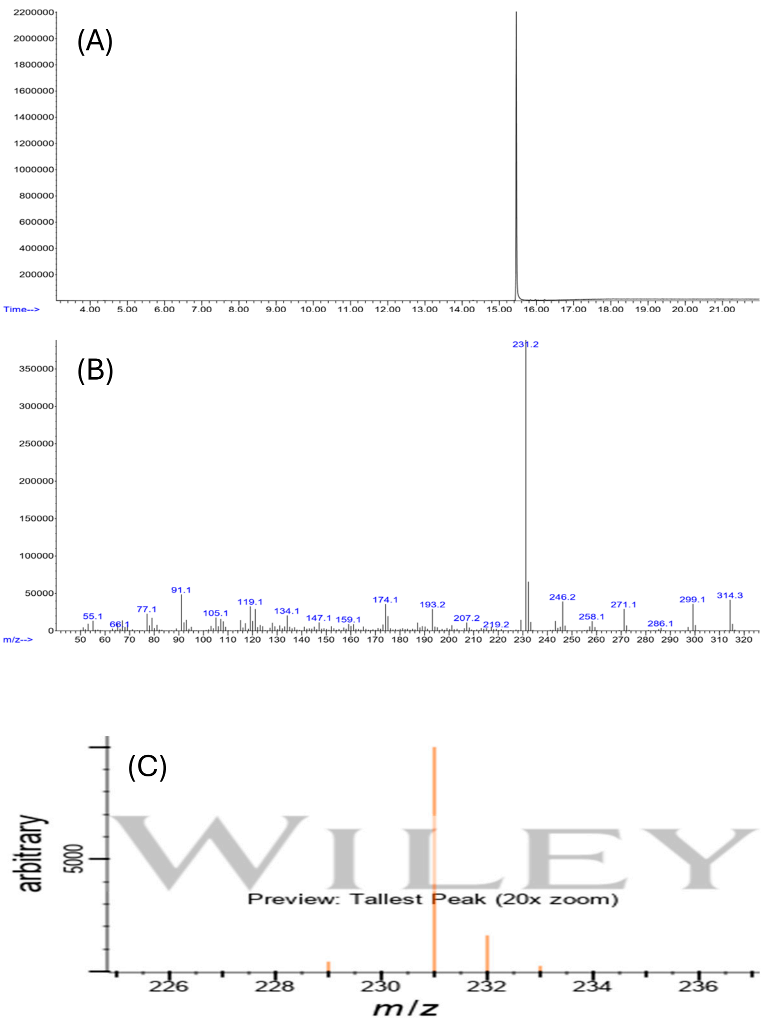

Figure S7. GC–MS analysis of CBDA
(**A**) Total ion chromatogram (TIC) of the CBDA sample analyzed in this study.
(**B**) Experimental electron ionization (EI) mass spectrum (70 eV) corresponding to the peak at RT = 15,458 min.
(**C**) Reference EI mass spectrum of CBDA obtained from the Wiley spectral library via PubChem [PubChem3]

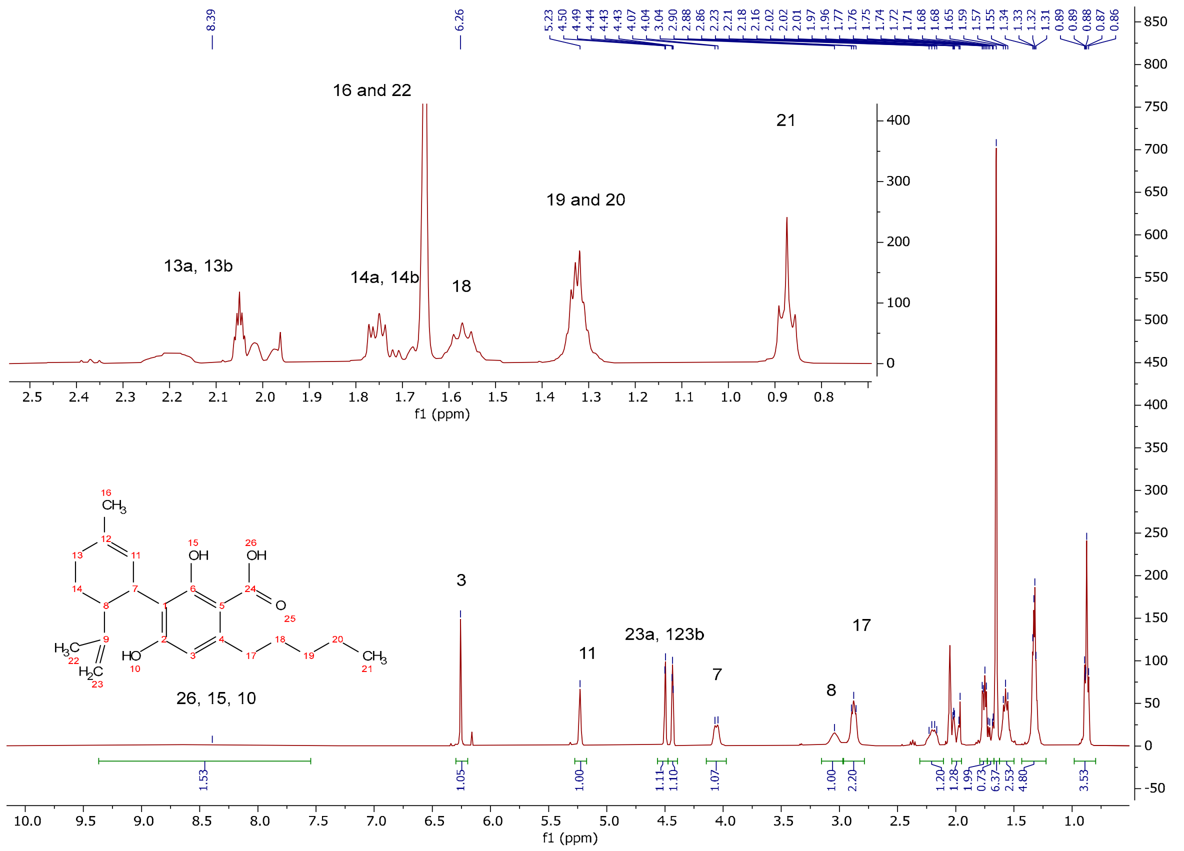

Figure S8. ^{1}H NMR spectrum of CBDA

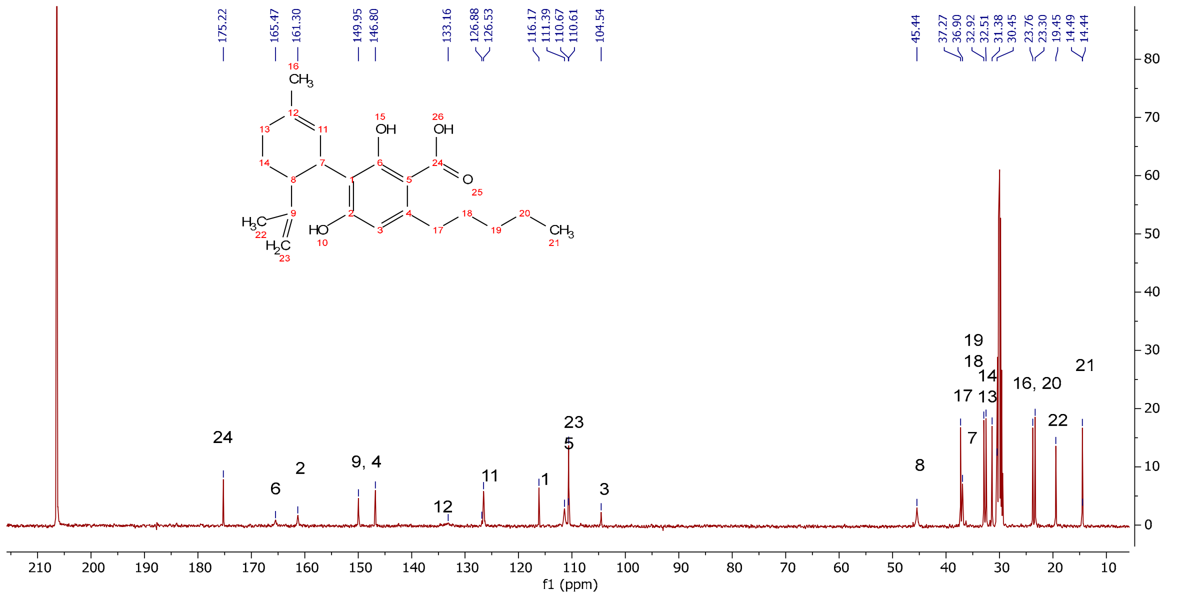

Figure S9. ^{13}C NMR spectrum of CBDA

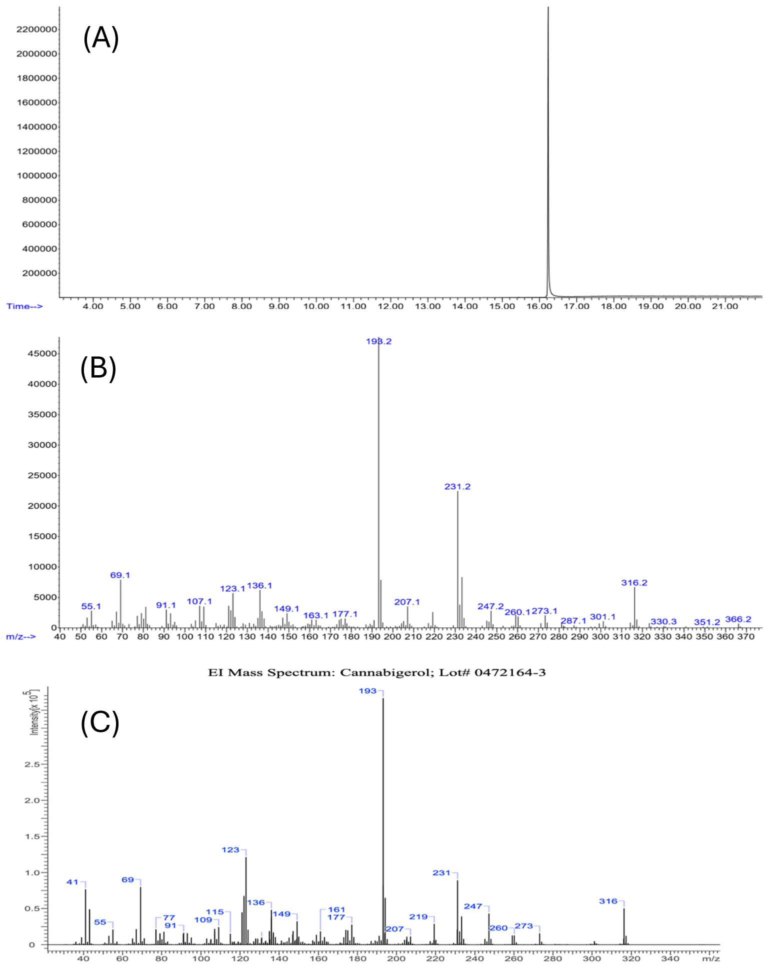

Figure S10. GC–MS analysis of CBG

(**A**) Total ion chromatogram (TIC) of the CBG sample analyzed in this study.

(**B**) Experimental electron ionization (EI) mass spectrum (70 eV) corresponding to the peak at RT = 16,236 min.

(**C**) Reference EI mass spectrum of CBG obtained from the SWGDRUG Monograph [SWGDRUG4].

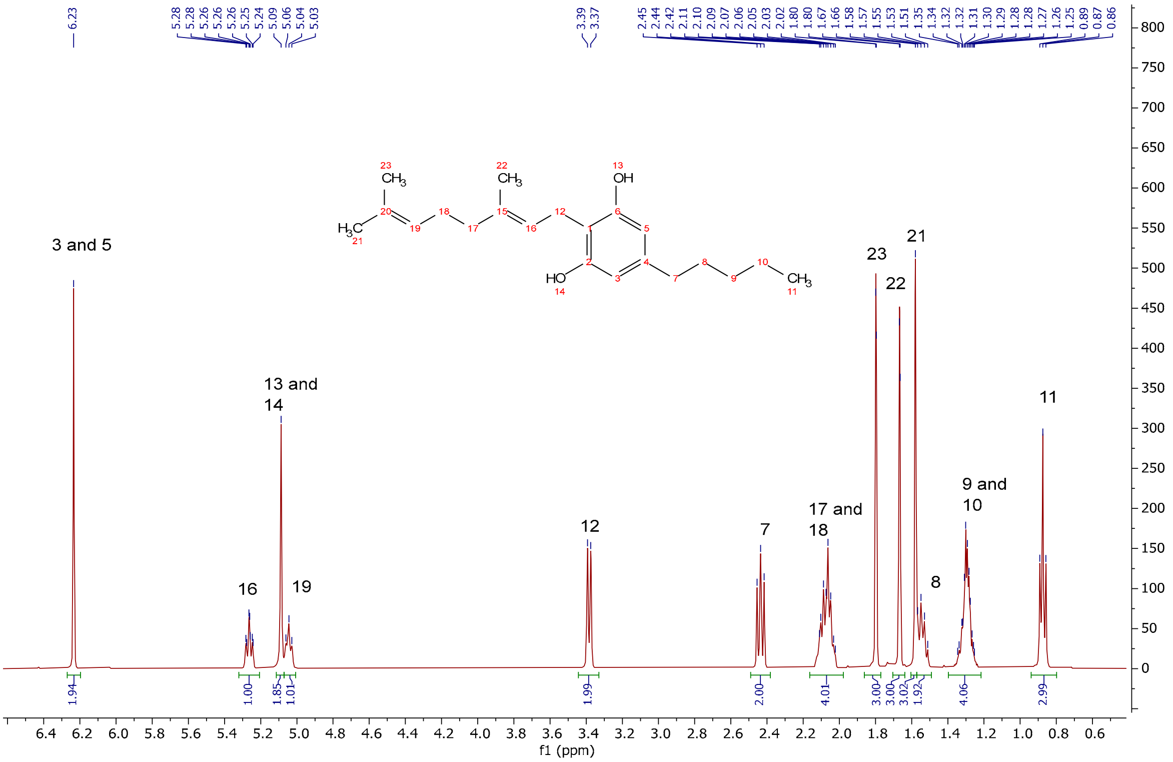

Figure S11. ^{1}H NMR spectrum of CBG

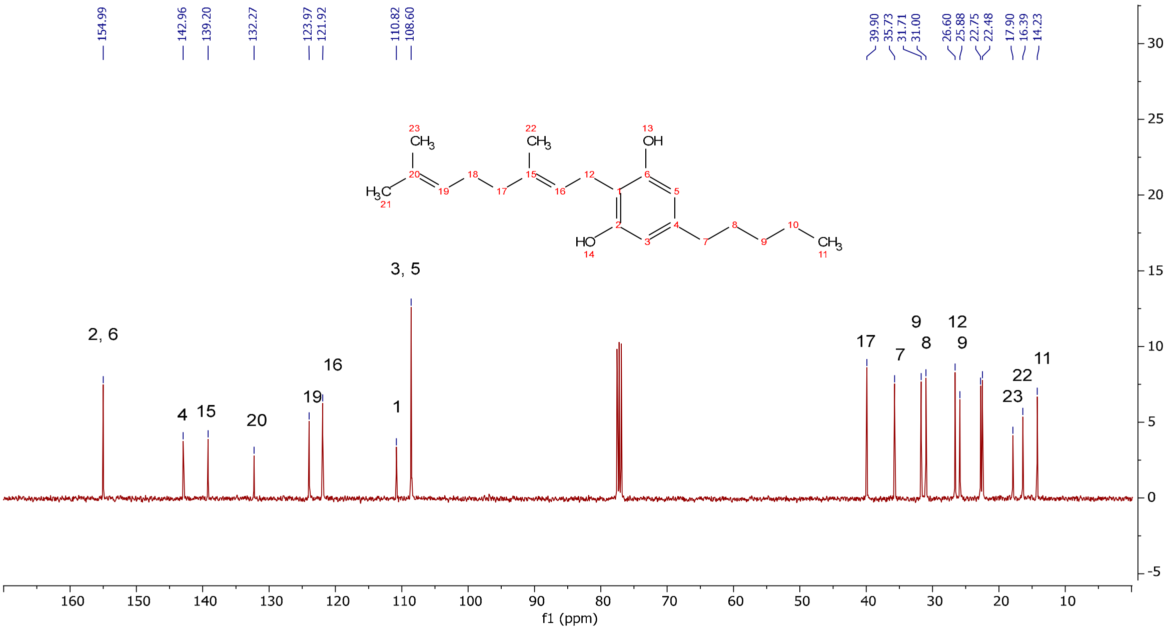

Figure S12. ^{13}C NMR spectrum of CBG

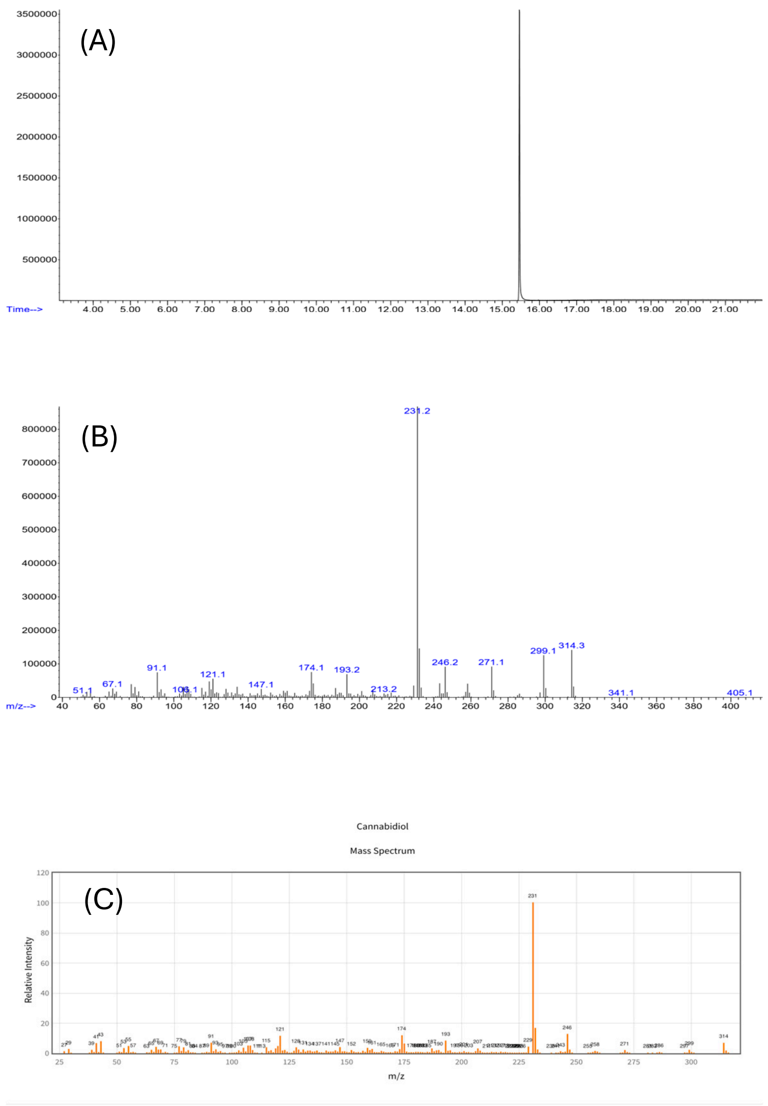

Figure S13. GC–MS analysis of CBD

(**A**) Total ion chromatogram (TIC) of the CBD sample analyzed in this study.

(**B**) Experimental electron ionization (EI) mass spectrum (70 eV) corresponding to the peak at RT = 15,458 min.

(**C**) Reference EI mass spectrum of CBD obtained from the NIST Chemistry WebBook [NIST2].

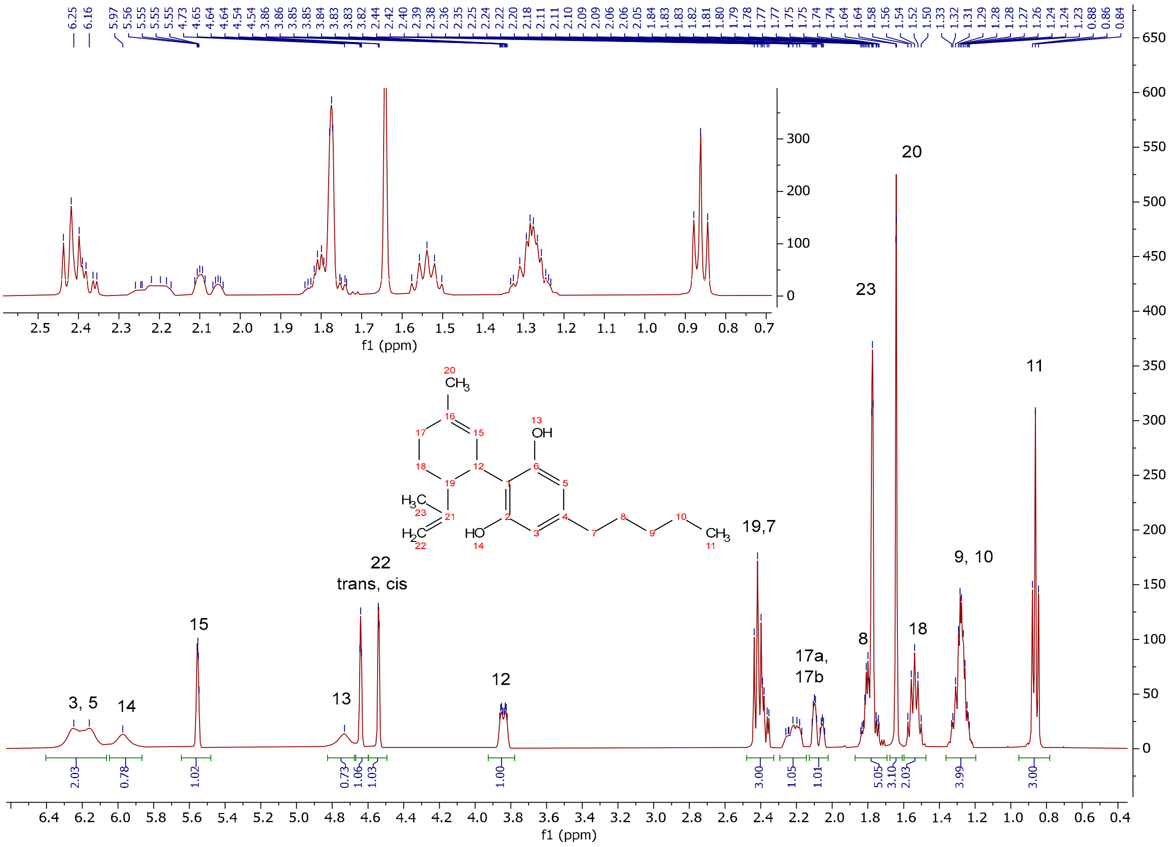

Figure S14. ^{1}H NMR spectrum of CBD

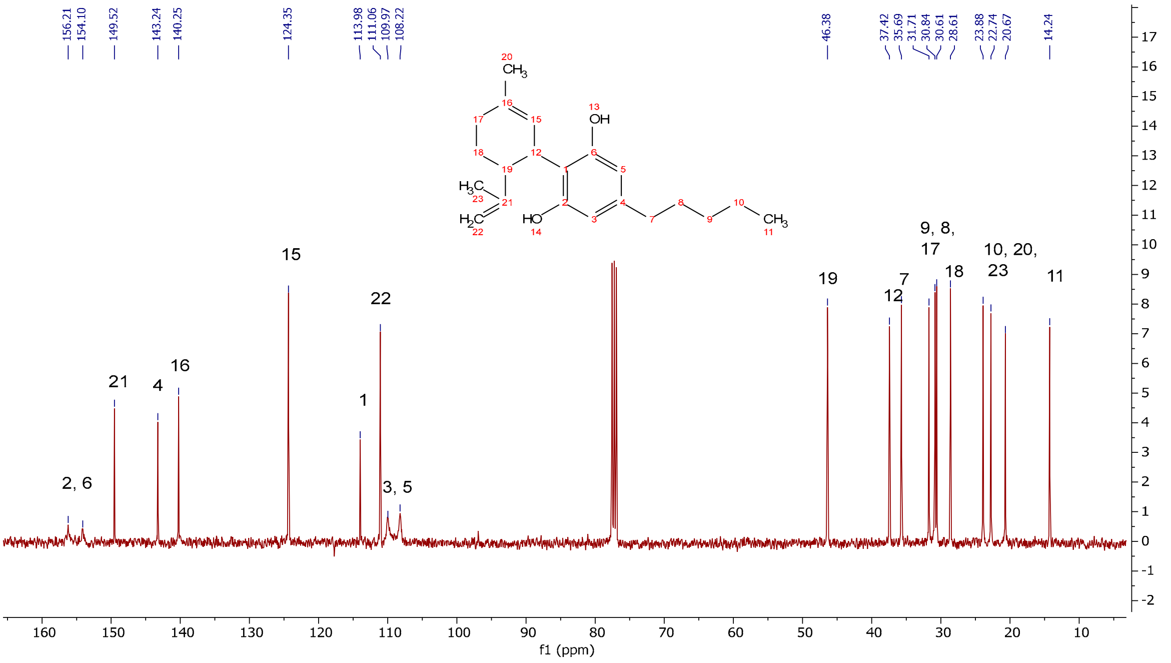

Figure S15. ^{13}C NMR spectrum of CBD

Table S1. Comparative antiradical activities of pure CBN and CBN-loaded liposomes against DPPH, hydroxyl, and superoxide radicals. Data are presented as mean ± SD (n = 3 independent experiments).

	**DPPH**	**Hydroxyl**	**Superoxide**
**CBN**	90.5 ± 2.9	82.0 ± 2.8	86.8 ± 2.9
**CBN-containing liposomes**	69.9 ± 3.4	49.9 ± 2.4	61.9 ± 3.1

References

59. NIST Chemistry WebBook, SRD 69; National Institute of Standards and Technology: Gaithersburg, MD, USA.

60. Cayman Chemical. GC–MS Spectral Data Sheet for Cannabidiol Diacetate (CAS 58452-86-9).

61. PubChem. Cannabidiolic Acid (CBDA); CID: 6440999. Spectral Information (Wiley Registry of Mass Spectral Data).

62. Scientific Working Group for the Analysis of Seized Drugs (SWGDRUG). Monograph: Cannabigerol (CBG).

63. NIST Chemistry WebBook, SRD 69; National Institute of Standards and Technology: Gaithersburg, MD, USA. Cannabidiol (CAS 13956-29-1).
